# Extracellular vesicles in malaria: proteomics insights, *in vitro* and *in vivo* studies indicate the need for transitioning to natural human infections

**DOI:** 10.1128/mbio.02304-24

**Published:** 2025-01-27

**Authors:** Núria Sima, Alberto Ayllon-Hermida, Carmen Fernández-Becerra, Hernando A. del Portillo

**Affiliations:** 1 ISGlobal, Barcelona, Spain; 2 IGTP, Germans Trias i Pujol Research Institute, Badalona, Barcelona, Spain; 3 School of Medicine and Health Sciences, University of Barcelona, Barcelona, Spain; 4 CIBERINFEC, ISCIII-CIBER de Enfermedades Infecciosas, Instituto de Salud Carlos III, Madrid, Spain; 5 Catalan Institution for Research and Advanced Studies (ICREA), Barcelona, Spain; Instituto Carlos Chagas, Curitiba, Brazil

**Keywords:** malaria, extracellular vesicles, molecular cargo, pathophysiology, diagnosis, biomarkers, vaccines, key gaps

## Abstract

Globally, an estimated 2.1 billion malaria cases and 11.7 million malaria deaths were averted in the period 2000–2022. Noticeably, despite effective control measurements, in 2022 there were an estimated 249 million malaria cases in 85 malaria-endemic countries and an increase of 5 million cases compared with 2021. Further understanding the biology, epidemiology, and pathogenesis of human malaria is therefore essential for achieving malaria elimination. Extracellular vesicles (EVs) are membrane-enclosed nanoparticles pivotal in intercellular communication and secreted by all cell types. Here, we will review what is currently known about EVs in malaria, from biogenesis and cargo to molecular insights of pathophysiology. Of relevance, a meta-analysis of proteomics cargo, and comparisons between *in vitro* and *in vivo* human studies revealed striking differences with those few studies reported from patients. Thus, indicating the need for rigor standardization of methodologies and for transitioning to human infections to elucidate their physiological role. We conclude with a focus on translational aspects in diagnosis and vaccine development and highlight key gaps in the knowledge of EVs in malaria research.

## INTRODUCTION

Roughly 3.2 billion people inhabit regions susceptible to malaria transmission. In 2022, there were approximately 249 million malaria cases globally across 85 endemic countries, marking a rise of 5 million cases from 2021 (1). Malaria deaths in the WHO African Region declined from 808,000 in 2000 to 548,000 in 2017, then rose to 604,000 in 2020, before dropping again to 580,000 in 2022. The malaria mortality rate decreased by 60% between 2000 and 2019, from 143 to 57 deaths per 100,000 population at risk, and fluctuated to 61 in 2020 and 56 in 2022. In the WHO South-East Asia Region, malaria deaths decreased by 77%, from around 35,000 in 2000 to 8,000 in 2022, with India and Indonesia contributing to 94% of all malaria deaths in the region (1). These data indicate that human malaria remains a major global health burden.

Five plasmodial species cause human malaria, with *Plasmodium falciparum* considered the deadliest, *P. vivax* the most widespread, with underestimated severe disease, *P. ovale* and *P. malariae* causing less significant morbidity and the simian parasite *P. knowlesi*, recently identified as a zoonotic disease from monkeys, whose global impact remains uncertain (2).

*Plasmodium* spp. undergo a complex life cycle involving female *Anopheles* mosquitoes and vertebrate hosts (3). Sporozoites are injected into the host dermis during a mosquito bite and enter the bloodstream, while some remain in the skin and trigger a host immune response (4). Sporozoites that enter the bloodstream migrate to the liver, invading hepatocytes and developing into liver stages. After replication, merozoites are released into the bloodstream, invading erythrocytes and initiating asexual development. This process includes three morphological stages: the ring, trophozoite, and schizont stages. Following each invasion, parasites make a developmental decision to either replicate asexually or differentiate into sexual stages termed gametocytes. The sexual cycle begins when gametocytes are ingested by a mosquito during a blood meal, leading to fertilization, formation of ookinetes, and eventual sporozoite release from the mosquito salivary glands (3).

Remarkably, *P. vivax* is one of the species that has evolved a dormant liver stage known as the hypnozoite (5), which upon reactivation causes clinical relapses and was thought to be the sole latent cryptic stage of this human malaria. However, more recent evidence has shown that *P. vivax* also evolved cryptic erythrocytic niches in the bone marrow (6, 7) and the spleen (8–10), the latter representing more than 95% of the total parasite biomass during asymptomatic chronic infections. *P. falciparum* parasites are also found in the bone marrow (11, 12), and the spleen (9). Even though molecular and clinical insights of these cryptic erythrocytic niches are presently very limited, they need to be incorporated into the life cycle of human malaria (Fig. S1). Moreover, as they are present during chronic asymptomatic infections, unveiling molecular mechanisms on their formation is needed if malaria elimination is to be achieved.

Extracellular vesicles (EVs) constitute a variety of double-membrane nanoparticles secreted by live cells across all three domains of life playing crucial roles in intercellular communication (13). These vesicles are historically and broadly categorized into exosomes and microvesicles (MVs), distinguished by their size, biogenesis, and composition (14, 15). Exosomes, ranging from 30 to 150 nm, originate from multivesicular bodies via inward budding, while MVs, ranging from 100 and 1,000 nm (16), bud directly from the plasma membrane. However, it is now acknowledged that exosomes and MVs are part of a more complex nanoparticles´ scenario now included in small and large EVs, respectively (17). As these terms are presently operational, we will continue using exosomes and MVs throughout this review acknowledging this limitation.

Both exosomes and MVs carry markers indicative of their parent cells and exhibit a selective cargo of molecules associated with their formation pathway (18). Exosome markers include, among others, tetraspanins (CD63, CD9, CD81), the endosomal sorting complexes required for transport (ESCRT) subunits and associated proteins, tumor susceptibility gene 101 (Tsg101), programmed cell death 6-interacting protein (Alix), and syntenin-1. Meanwhile, typical MV markers are Annexin A1 and ADP-ribosylation factor 6 (ARF6) (16, 18). Importantly, some markers have been used to identify the cell source of microparticles (MPs) in malaria natural infections, i.e., cluster of differentiation (CD), CD41 for platelets, CD105, CD51, and CD144 for endothelial cells, CD235a for RBCs, CD45 for leukocytes, CD11b for monocytes, and CD3 for lymphocytes (19, 20). Additionally, CD71 can be used as a reticulocyte-derived EV marker to study EVs originating from the host cell of *P. vivax* (21–24). Research on these vesicle types, derived from both host and parasite sources, has unveiled their significant involvement in disease pathogenesis, susceptibility, intercellular signaling, and immune responses (25, 26).

Research on EVs holds significant potential as new therapeutic agents and diagnostic tools (13). The role of EVs in modulating immune responses was first described in a pioneering study using exosomes secreted by a human B-cell line (27). Since then, the immune modulatory properties of exosomes and MVs from other cells have been demonstrated (25, 28). Notably, exosomes used in human clinical trials against late-stage cancer patients have shown great potential as cell-free vaccines (29). Although no clinical trials for parasitic diseases have been reported yet, original proof-of-concept studies have demonstrated their potential as novel vaccines for *Toxoplasma gondii* (30), *Leishmania major* (31), and *P. yoelii* (32), where EVs from antigen-loaded dendritic cells or infected cells protected animal models from infection.

EVs are present in all biological fluids, and their molecular composition from various origins and pathologies already demonstrates their immense potential as biomarkers (33, 34). However, these studies are currently challenged by the lack of consensus definitions and protocols for isolating and characterizing EVs from different cell and tissue origins. Overcoming these technical difficulties, as recently described for parasitic diseases (35, 36), will pave the way to unveil the molecular basis of pathophysiology and to use EVs as novel diagnostic tools and therapeutic agents against parasitic diseases.

**Fig 1:**
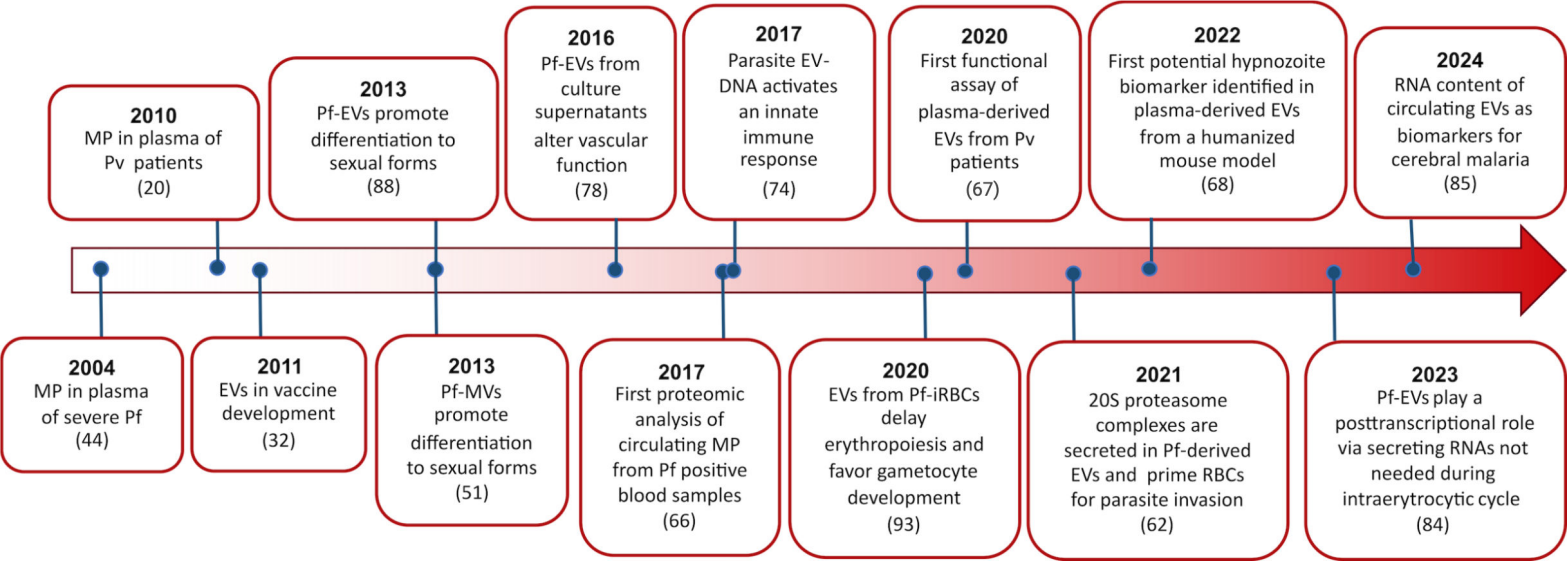
Timeline of the major milestones in extracellular vesicle research in the field of malaria.

In this review, through an extensive revision and proteomics meta-analysis of previously available publications of EVs and malaria, we aimed to reveal insights into EV biogenesis, specific cargo, and role in pathophysiology and host-parasite interactions, with a focus on comparisons of *in vitro*, *in vivo,* and human studies. Last, we will review the perspectives of EVs in malaria as novel disease biomarkers and therapeutic agents. Figure 1 presents a historical overview of key research findings on EVs in malaria.

## BIOGENESIS, COMPOSITION, AND UPTAKE OF EVs

### Biogenesis

EV biogenesis involves complex and highly regulated processes occurring in various cell types. Exosome formation begins with the inward budding of endosomal membranes, resulting in the formation of multivesicular bodies (MVBs), which later fuse with the plasma membrane to release the exosomes into the extracellular space (21, 22, 37). Exosome biogenesis is orchestrated by the ESCRT machinery, which selectively includes ubiquitinylated cargo (38). However, alternative ESCRT-independent mechanisms, such as those involving tetraspanins or ceramides, have also been reported (39–41). The fusion of the MVB to the plasma membrane for exosome release is driven by several proteins of the Rab-GTPase family, some of which are common with the secretory autophagy pathway. In addition, other protein complexes that regulate vesicular trafficking, including soluble N-ethylmaleimide-sensitive factor attachment protein receptor (SNARE) and ESCRT are also shared between autophagy and exosome biogenesis, underscoring the interconnected nature of these cellular processes (42). On the other hand, MVs are produced directly by the outward budding of the plasma membrane. The ESCRT machinery acts at different points during MV formation, and this process is regulated by cytoskeleton rearrangement, lipid redistribution, and the local accumulation of certain signaling molecules like calcium (14, 37, 43).

Recent studies highlight that EV biogenesis is modulated by cellular conditions, stress, and specific molecular signals, including those driven by metabolic and inflammatory pathways (37). Lipids, proteins, and RNAs packaged into EVs are selectively sorted and reflect the functional state of the parent cell, making EVs critical players in intercellular communication (14, 37), however, the mechanisms by which cells selectively package the specific cargo remain unclear. Understanding EV biogenesis’s regulatory mechanisms is key to unraveling their roles in physiological and pathological contexts.

Initial observations in malaria-infected individuals revealed elevated levels of circulating EVs associated with disease severity (20, 44, 45). These EVs are now known to be released by parasites, infected red blood cells (iRBCs), and other host body cells (35, 46, 47). However, the processes involved in EV formation from parasites and iRBCs are poorly understood. Additionally, it is unclear what triggers the production of EVs by host cells in response to *Plasmodium* infection. Despite lacking the canonical vesicular trafficking pathway, iRBCs still release EVs (48). The ESCRT machinery, particularly the ESCRT-III subcomplex, facilitates EV formation in higher eukaryotes (49). In *P. falciparum*, an operational ESCRT-III machinery exists, activated through an alternative recruitment pathway orchestrated by PfBro1 and involving PfVps32/PfVps60 proteins. This machinery plays a pivotal role in the biogenesis of exosomes and MVs in iRBCs (48). Further research is needed to understand the biogenesis pathways of EVs and their role in the progression of malaria.

### Composition

EVs derived from iRBCs with distinct asexual phases of the malaria parasite exhibited differing levels of EV markers compared to uninfected (u)RBCs (50). EVs derived from *in vitro* cultured *P. falciparum*-iRBCs (*Pf*-iRBCs) are released during all stages of the asexual cycle and originate from specific subdomains of the iRBC (51–54).

#### Lipids

The lipid bilayer of EVs is primarily composed of cholesterol, sphingolipids, and phospholipids (55). Additionally, they may carry other lipids, such as fatty acids, prostaglandins, or leukotrienes, either within the lumen or on the membrane. Moreover, numerous studies have reported that the lipid composition of EVs is specific to their cell type and site of origin (55). Interestingly, it has been shown that iRBC MVs have a distinct lipid profile compared to uRBC MVs, being particularly enriched in phosphatidylserine (PS) and phosphatidylinositol (PI) and at the expense of phosphatidylcholine that is reduced compared to the RBC membrane (45, 51, 56, 57). It has been suggested that MVs originate by budding from specific plasma membrane microdomains, potentially from lipid rafts, which in iRBCs are also enriched in PS (51, 58, 59). Furthermore, iRBC-derived MVs were shown to be enriched with various sphingolipids, including ceramide, lactosylceramide (LacCer), dihydroceramide (dhCer), and GM3 gangliosides (56). Among these, ceramide, LacCer, and GM3 are recognized as potent bioactive lipids that play critical roles in modulating immune responses (60, 61). The presence of these lipid species in iRBC MVs may be pivotal to the immunomodulatory properties attributed to *P. falciparum-*iRBC MVs (51), further highlighting their potential role in parasite-host interactions (62).

#### Proteins

Malaria EVs harbor proteins associated with the membranes of RBCs, including components of the Maurer’s clefts (SBP1, Rex1/2, MAHRP1/2, PfMC-2TM), proteins linked to the surface membrane of RBCs (Clag3.1/2, RESA and MESA), and proteins associated with the membrane of the parasitophorous vacuole membrane (Exp-2, Etramp2) (52). Moreover, they contain proteins crucial for parasite invasion, such as erythrocyte-binding antigens (EBA-175, EBA-181), which bind to glycophorins during merozoite invasion, and rhoptry proteins (RhopH2/H3 and Rap2) (51, 52). Notably, certain protein families in EVs from the late-stage of *Pf*-iRBC, such as PHIST and Rifin, but not PfEMP1 and Stevor, suggest selective loading mechanisms. In addition, EVs released during the early asexual stages of the parasite carry PfEMP1 and trigger a mild cytokine response and transcriptional alterations in human monocytes, partly relying on PfEMP1 (53). The transfer of PfEMP1 through EVs may play a crucial role in shaping the intricate interactions between the parasite and the host’s immune system during infection.

A proteomic analysis of EVs derived from *P. falciparum in vitro* culture demonstrated the presence of parasite proteins in *Pf*-iRBC-derived EVs across various parasite stages, with human proteins being specific to EV types, i.e. MVs and exosomes (54). Additionally, most human proteins are shared among different parasite strains, albeit with varying expression levels, with only 10% being strain-specific. Furthermore, invasion-associated proteins of *P. falciparum*, such as AMA-1, reticulocyte binding protein, MSP-1, and EBA-175, are conserved across different parasite strains. Interestingly, it was found that invasion effectiveness was reduced in the presence of a large number of *Pf-*iRBC-derived MVs, but not with *Pf-*iRBC-derived exosome preparations, in both, non-virulent and virulent *P. falciparum* strains (54). These findings suggest that variations in *P. falciparum* strain virulence may be attributed to selective loading and distinct cell-specific proteins in EVs, and highlight the specific functions of different EV sizes on parasite invasion.

Another study from *P. falciparum in vitro* culture successfully identified various subpopulations of EVs released by *Pf*-iRBCs, differing in size, protein content, and mechanical properties of the membrane. Smaller EVs (10–70 nm) are enriched in proteins related to the complement system, while larger EVs (30–300nm) contain functional 20S proteasome complexes (EV-20S) (62, 63). These findings suggest distinct roles for different EV subtypes in parasite-host interactions. Furthermore, *Pf*-iRBC-derived EVs are enriched in parasitic and host kinases that promote the phosphorylation and degradation via EV-20S complexes of cytoskeletal proteins of the RBC cortex (i.e. β-adducin, ankyrin-1, dematin, and Epb4.1), enhancing host cell membrane deformability and disrupting cytoskeletal integrity (62). Importantly, smaller and larger EVs exhibit differential capabilities in fusing with early endosomes, implying distinct cellular targets (63). Additionally, glycophorin A, a receptor for *P. falciparum* EBA-175 (64), and several channels in RBC membranes are phosphorylated upon stimulation by *Pf*-iRBC-derived EVs (62). However, the significance of these post-translational modifications in parasite invasion remains to be elucidated.

Discrepancies in EV size and composition across studies may result from differences in isolation methods or sample sources. In a study of experimental cerebral malaria (CM) in mice infected with *P. berghei*, circulating MVs were enriched in proteins with potential roles in malaria pathogenesis, such as carbonic anhydrase 1 (CA-I) and myeloid-related protein 8 (MRP-8) (65). Moreover, circulating MVs from *P. falciparum* patients contain more proteins than those in healthy donors (HD) and were enriched in proteins related to inflammation, complement and coagulation, adhesive proteins and receptors, and hemoglobin (66). Studies on EVs from *P. vivax* natural infections and humanized mouse models have identified several malaria antigens including merozoite surface proteins MSP7 and MSP9, Serine-repeat antigen 1, and HSP70 with the potential for vaccine development and possible biomarkers of hypnozoite infection (23, 67, 68). Of interest, MSP1, MSP3, and pHISTc, all immunogenic in natural infections, were detected in the proteomic analysis mentioned above employing various purification techniques and sample origins (23, 67, 68). Moreover, EVs from HD plasma samples contain more human proteins than those from *P. vivax*-infected patients, suggesting a selective sorting mechanism of the human protein cargo during *P. vivax* infections (67).

To deepen our understanding of human malaria virulence mechanisms, further analysis of EV contents from diverse experimental infections, clinical isolates, patient samples, and functional validation of these components is crucial.

##### Meta-analysis

**Fig 2:**
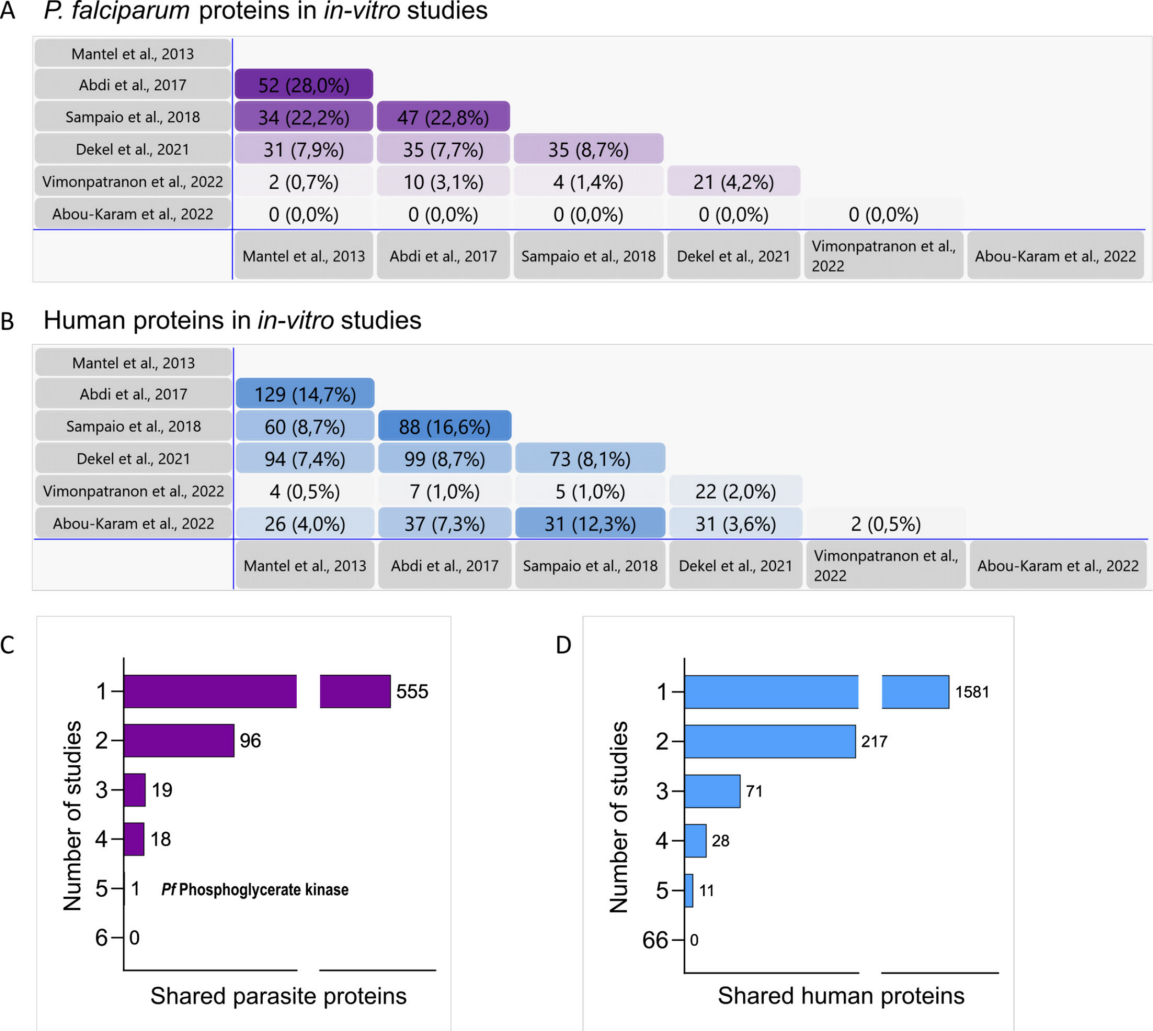
Meta-analysis compiling data from six studies investigating the protein composition of extracellular vesicles derived from supernatants of *Plasmodium falciparum*-infected red blood cell cultures. (A and B) Pair-to-pair comparison of proteins found in EVs in six independent studies. The horizontal and vertical axes display the authors of the studies to show all possible pairwise combinations. The percentages in brackets represent the proportion of matching proteins, calculated based on the number of proteins identified in each study pair. (A) *P. falciparum* proteins. (B) Human proteins. (C and D) Bar graphs indicate the cumulative number of proteins shared among the six *in vitro* studies. (C) *P. falciparum* proteins. (D) Human proteins. Pairwise comparisons were conducted using FunRich (69) and were based on UniProt accession numbers.

This review conducted a meta-analysis of seven distinct studies examining the protein composition of EVs derived from either plasma of *P. falciparum*-infected patients (66) or parasites cultured *in vitro* (51–54, 62, 63) (Fig. 2) (Tables S1 to S5). This analysis helped in identifyin common patterns and potential shared biomarkers in EV studies while also evaluating the consistency of proteomic findings across various methods and clinical or experimental settings. We also compiled a table listing all parasite proteins identified through proteomic analysis for *P. vivax* (Table S6).

### Methodology

The protein lists from each study (51–54, 62, 63, 66) were obtained from the main tables, supplemental information, or, when available, the ProteomeXchange database (Table S1). Based on UniProt accession numbers, pairwise comparisons were conducted using FunRich (69) and the Venn web tool (https://bioinformatics.psb.ugent.be/webtools/Venn/). The complete list of proteins and detailed analysis can be found in Tables S2 to S5.

### Comparison of *in vitro* studies

Figure 2 presents the number of matching proteins identified when comparing each *in vitro* study pairwise (51–54, 62, 63). Shared proteins ranged from 0% to 28% for parasite proteins and from 0.5% to 16.6% for human proteins (Fig. 2A and B). A closer examination of the *P. falciparum* protein subset revealed that 555 parasite proteins, out of the 689 identified across six studies, were exclusive to a single study (Fig. 2C). Among the remaining proteins, 18 were identified in four studies, though not all in the same set of four works; instead, these proteins appeared in various overlapping combinations of four studies (Tables S2 and S4). Similarly, 19 proteins were observed in three works, but again, in different sets of studies. Additionally, 96 proteins were identified in two investigations, although in different pairs. Finally, one protein, phosphoglycerate kinase, was consistently identified in five studies whereas none was identified in the six studies. The specific details of each comparison, including which protein was identified in which study, are provided in Tables S2 and S4.

Of interest, proteins shared by a minimum of three studies encompass invasion-associated proteins (such as MSP-1, MSP-9, glycophorin binding protein 130, 14-3-3 protein I, rhoptry proteins, and rhoptry-associated proteins), molecules involved in nucleic acid and protein biosynthesis (including S-adenosylmethionine synthetase, DNA/RNA-binding protein Alba, elongation factor 1-alpha, and various ribosomal proteins), as well as proteins linked to gametocyte development, maturation and transmission to mosquitoes (such as phosphoethanolamine N-methyltransferase) (Tables S2 and S4). These results underscore the role of EVs in enhancing the parasite’s virulence and facilitating its transmission. Furthermore, *P. falciparum* lactate dehydrogenase (PfLDH), a well-established biomarker and indicator of parasite biomass (70), was detected in EVs from culture supernatants in four studies, reinforcing the diagnostic prospects associated with EVs (Tables S2 and 4).

A comparison of the human proteins identified in EVs from parasite *in vitro* culture supernatants (51–54, 62, 63) revealed, similar to the *P. falciparum* identified proteins, that out of 1908 proteins identified, 1581 were detected in a single study, 11 proteins were found in five research works, 28 were commonly detected in different sets of four studies, 71 were shared by three different combinations of studies, and 217 were identified in two studies in different pairs. Again, no common proteins were identified in all studies based on accession numbers (Fig. 2D) (Tables S3 and S4).

Glycophorins, Band 3, and spectrin are proteins typically found in extracellular vesicle (EVs) from RBCs and were detected in at least one *in vitro* study, though with low overlap. However, common hemoglobin subunits are identified in at least five studies, while the heat shock 71 kDa protein, ankyrin, and GAPDH are reported in four studies (Tables S3 and S4). EVs are thought to act as post-translational regulators and may prime RBCs for new infections by modifying the host cell cytoskeleton (62). Notably, components of the 20S and 26S proteasome complexes were detected in the six studies analyzed in this review (Table S3), and cofilin-1, an enzyme crucial for cell morphology and cytoskeleton organization (71), was consistently identified in four studies (Tables S3 and S4), supporting this theory.

The notorious lack of overlap among these *in vitro* studies may stem from variations in EV isolation and purification methods, differences in parasite developmental stages, timing before processing and protocols for processing samples, freeze-thawing cycles, mass spectrometry sensitivities, *in silico* proteomics, and significant disparities in data availability. Therefore, this indicates the need for rigorously standardizing protocols for the analysis of the molecular cargo of EVs across different laboratories and for the mandatory need to facilitate raw data from open-access repositories.

### Comparison of *in vitro* and *in vivo* studies

From the 30 parasite proteins identified in EVs from patients, 16 were present in at least one *in vitro* study (51–54, 62). These encompass cytoskeletal, heat-shock, membrane-related proteins, and proteins involved in invasion and pathogenesis (Tables S2 and S5). Notably, 11 proteins were uniquely identified in one *in vitro* study. Two proteins −GTP-binding nuclear protein RAN/TC4 and Heat shock protein 70− were shared between two *in vitro* studies and the *in vivo* data, while one protein—enolase—appeared in three studies. Additionally, actin and tubulin were consistently detected across two sets of four studies (Tables S2 and S5).

Of the 47 human proteins identified in EVs from the plasma of *Pf*-infected patients (66), 26 were also found in at least one *in vitro* culture study (51–53, 62) (Tables S3 and S5). Of these, 14 proteins appeared in only one independent *in vitro* study and 10 were detected in no overlapping sets of two studies. Only two proteins—spectrin alpha and beta—were shared across three *in vitro* studies and the *in vivo* data. In addition, no single protein was found in all groups. Proteins identified included RAB, cytoskeletal, inflammation-related, and complement proteins (Tables S3 and S5).

The lack of overlap between samples obtained from *in vitro* studies as opposed to samples obtained directly from patients might simply reflect the limit of detection of parasite proteins in the complex mix of plasma-derived EVs. Indeed, parasite signals were significantly increased when reticulocyte-derived EVs from infections were immunocaptured (23) as opposed to isolate them by size (67). Regardless, this proteomic meta-analysis highlights that the molecular cargo obtained from EVs derived from *Pf*-iRBC cultures differs dramatically among different studies and may not accurately reflect the EV composition found *in vivo*. Thus, underscoring the need for transitioning to studies in patients to accurately determine the molecular cargo of circulating EVs in natural infections.

#### Nucleic acids

*Pf*-iRBC-derived EVs contain host and parasite RNA (72, 73) and genomic (g)DNA (74). The RNA composition of *Pf*-iRBC-derived EVs consists of approximately two-thirds human-origin RNA and one-third parasitic-origin RNA, with elevated levels of host micro (mi)RNAs and parasite transfer (t)RNAs (72, 73). In response to nutritional perturbations, ring-stage parasites increase the production of EVs with an altered expression pattern of these RNA biotypes (73), which could facilitate inter-parasite communication to relay information regarding environmental alterations. Apart from miRNAs and tRNAs, these EVs contain other human small regulatory RNAs like Y-RNAs, vault RNAs, small non-coding (sno)RNAs, and piRNAs (72, 73).

*Plasmodium* parasites are unable to produce miRNAs (75, 76), indicating that miRNAs found in EVs originate solely from the host. Interestingly, parasite antigens appear to influence the production of host miRNAs (6, 77), which subsequently regulate the expression of *P. falciparum* genes through intricate mechanisms (78, 79). The composition of miRNAs within EVs reflects that of the parent cell, suggesting that circulating EVs may serve as cellular miRNA status indicators, potentially influenced by host-pathogen interactions. EVs released from *Pf*-iRBCs have been identified as carriers of functional miRNA-Argonaute 2 (Ago2) complexes, where Ago2 is an essential component of the RNA-induced silencing complex (RISC) (78, 80). These complexes exhibit regulatory roles in gene expression both within iRBCs and in host endothelial cells. Specifically, within iRBCs, miR451/140-Arg2 complexes target and downregulate the expression of PfEMP1 (80). Moreover, upon internalization by endothelial cells, these complexes influence gene expression patterns and barrier properties (78). In addition, EVs transfer plasmodial RNA into endothelial cells, but their function is still under study (72). A targeted study of EV-derived microRNAs in Thai malaria patients found has-miR-15b-5p and has-miR-150–5p up-regulated in *P. vivax* patient blood and has-let-7a-5p up-regulated in both *P. vivax* and *P. falciparum* patient blood (81). However, the has-miR-150–5p finding was inconsistent in another study (82), suggesting that further validation would be required.

Within the EVs derived from *Pf*-iRBCs, a range of plasmodial RNAs have been identified, including messenger RNAs (mRNAs) that encode exported proteins and proteins linked to drug resistance, along with non-coding RNAs such as rRNAs, small nuclear RNAs (snRNAs), and tRNAs (72, 73). Notably, the mRNA content of EVs differs from the cellular transcriptome, implying a selective loading mechanism (83). It has been postulated that EVs could function as a post-translational RNA regulatory mechanism to maintain intracellular RNA levels. This is supported by the observation that EV transcriptomes derived from *Pf*-iRBCs exhibit periodic patterns shifted from those of the entire parasite, with peaks of expression in the whole parasite corresponding to troughs of RNA secretion via *Pf*-iRBC EVs, and vice versa (84). Moreover, studies on human patients have revealed that circulating EVs from CM patients are primarily enriched in mRNA originating from brain tissues, suggesting a potential association between EV transcriptome and disease progression (85).

### Uptake

EVs originating from *Pf*-iRBCs and those derived from other patient cells have been observed to fuse with the host cell membrane for cargo delivery (51, 62, 67, 86). Of particular note, Ofir-Birin et al. have introduced an imaging flow cytometry method to track the internalization of EV cargo within the cellular environment, providing valuable insights into the dynamics of EV cargo internalization and associated signaling pathways (86). This innovative approach holds significant potential for advancing our understanding of the mechanisms involved in EV-mediated cellular interactions. Furthermore, evidence suggests that *P. falciparum* exploits host sialylated N-glycans to facilitate the uptake of EVs by immune system cells (87). However, further investigation is warranted to ascertain whether this mechanism applies to other types of host cells.

## EV-MEDIATED CELL-TO-CELL COMMUNICATION

**Fig 3:**
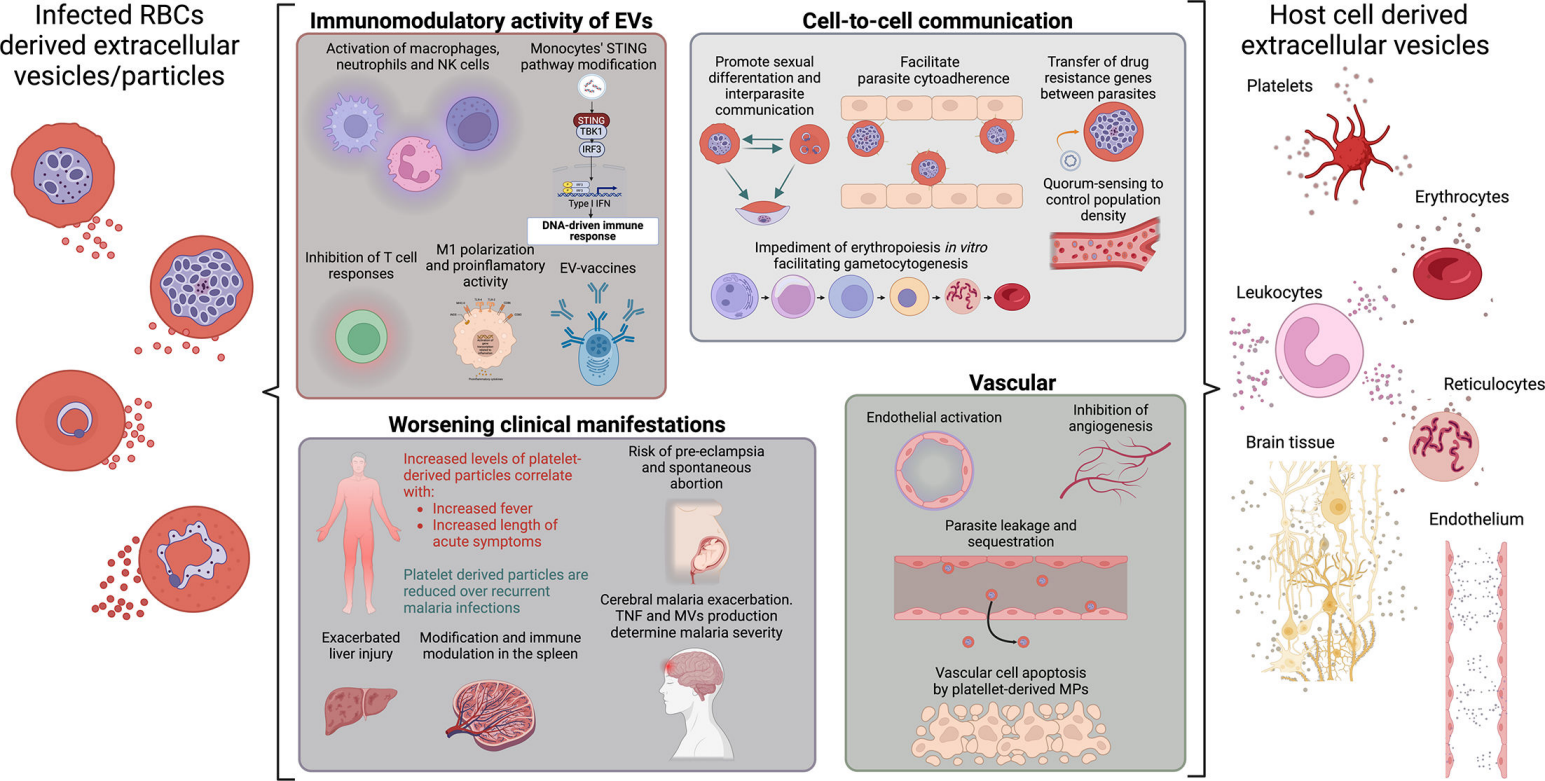
Overview of effects caused by extracellular vesicles derived from infected RBCs and host cells during malaria infection. EVs from iRBCs can produce a wide range of effects in many different cell types, tissues, and organs acting as key players in the intricate interactions between parasite and host. The figure highlights its role in immunomodulating the host response (45, 49, 53, 57, 74, 78), the vascular alterations (44, 74, 96), the worsening of the clinical manifestation (93, 97–100), and cell-to-cell communication (67, 78, 86, 88, 90, 91). In addition, human host cells may produce off-site effects through EVs as intercellular communicators during malaria infection. Host-derived EVs are capable of increasing symptomatology (20, 44, 101), especially fever, but also activate immune cells (44, 102, 103), increase severity (44, 97, 99, 104), produce apoptosis and endothelial activation (46), and increase the cytoadherence of the parasite (44, 97, 99). Created with BioRender.

EVs serve as critical mediators of intercellular communication, conveying their cargo from donor to recipient cells or engaging with receptors on target cell membranes to induce signaling pathways, even without internalization of the EVs (62, 64, 67, 72, 74, 78, 87–89). Notably, recent studies have shed light on the involvement of EVs in various cellular processes within the context of malaria infection (Table S7). EVs released by *Pf*-iRBCs have been implicated in facilitating communication within both the parasite and host cell populations (62, 74, 80, 86, 88). Initial observations indicated that parasite-conditioned medium facilitated the formation of gametocytes (90) with the added capability of inducing sexual conversion within controlled *in vitro* settings (91) (Fig. 3). Subsequent investigations have elucidated that MVs, released during late asexual erythrocytic stages, and exosome-like vesicles, generated during the early phases, play pivotal roles (51, 88). These vesicles are shown to be internalized by iRBCs, thereby fostering the generation of transmission stages of the parasite, probably by transferring either parasite or host factors (51, 88). Importantly, it was later demonstrated that EV-depleted conditioned medium used to culture P. falciparum retained the capacity to generate transmission stages, which was attributed to the presence of lysophosphatidylcholine in the medium (92). Furthermore, EVs released from *Pf*-iRBCs have been shown to impede erythropoiesis *in vitro*, potentially facilitating the development of various gametocyte stages within immature red blood cells before their release into circulation (93) (Fig. 3). While the exact role of EVs in the inhibition of erythroid development by *P. vivax* parasites remains to be fully elucidated (94), circulating EVs from *P. vivax*-infected patients (*Pv*EVs) have been implicated in signaling human spleen fibroblasts, promoting cytoadherence through NF-kB translocation to the nucleus and upregulation of ICAM-1, thus potentially contributing to the formation of splenic cryptic niches in vivax malaria (67, 95) (Fig. 3).

EVs have also been found to harbor DNA, capable of transferring it to other iRBCs or host cells, thereby potentially contributing to parasite virulence and modulating host immune responses (74, 88). Specifically, Regev-Rudzki et al. demonstrated the transfer of episomal DNA containing drug resistance genes between parasites via EVs in a trafficking protein 2 (PfPTP2)-dependent manner, highlighting the role of EVs in parasite virulence and communication (88). In parallel, Sisquella et al. revealed that genomic DNA released into human monocytes via EVs derived from the ring stage of the parasite’s life cycle activates the STING-dependent DNA sensing pathway, thus modulating host gene expression and triggering innate immune responses (74). Additionally, proteomic analysis of EVs released during the late stages of the parasite’s life cycle has revealed the presence of plasmodial antigens and invasion-related parasite proteins (52). Immunogenic proteins have also been identified in EVs from vivax infections (23). These findings support the idea that EVs play a role in antigen presentation and modulation of host immune responses during malaria infection (Fig. 3). This highlights their potential advantage in the development of EV-based vaccines. Studies in mice have revealed that *P. berghei* iRBC-EVs promote the release of the tumor necrosis factor (TNF) and activate macrophages (57). In the same line, several investigations in humans showed that *Pf*-iRBC-EVs are taken up by human monocytes and induce the cellular immune response (74, 86). Moreover, EVs from a culture-adapted severe malaria strain produced a more marked induction of monocytes than non-severe strains, although the effect is not linked to differences in the miRNA content, suggesting that miRNAs might not be a significant factor during monocyte polarization (105). Interestingly, Gualdrón et al. showed that in vivax malaria, plasma-derived EVs from patients interact more with human spleen cells such as T cells, monocytes, B cells, and erythrocytes compared to EVs from healthy donors (96), suggesting a role of *Pv*EVs in immune modulation in the spleen.

Finally, a role in quorum-sensing has been proposed in which *Pf-*iRBC EVs act as signaling molecules in response to the cell population density since EVs derived from high parasite density cultures regulate the parasite population (53). Collectively, these findings underscore the multifaceted roles of EVs in host-parasite interactions during malaria (Fig. 3).

## EVs IN MALARIA PATHOLOGY

### Vascular dysfunction and EVs

In the complicated context of vascular dysfunction during malaria infection, MVs and exosomes emerge as key players, shaping endothelial responses and influencing the delicate balance of vascular homeostasis. Valuable insights were revealed by observing a notable elevation in RBC MVs in *P. falciparum* malaria patients, which directly correlated with disease severity and vascular dysfunction (45). Further investigations have deepened into the effect of MVs, demonstrating that malaria-induced vascular dysfunction is accompanied by inflammatory responses, with EVs derived from iRBCs carrying miRNA-Ago2 complexes capable of modulating target genes (78). When these EVs are internalized by endothelial cells, they contribute to altered barrier properties, increased pro-inflammatory cytokine production, and vascular dysfunction, promoting endothelial activation, leakage, and parasite sequestration, as well as pathology during malaria infection, particularly in severe malaria cases (78). Moreover, exosomes from *Plasmodium*-infected hosts inhibit VEGFR2 expression and hinder angiogenesis *in vivo* (104) (Fig. 3).

Of interest, recent data indicate that non-EV particles and nanoparticles, including products of hemoglobin degradation and heme-laden nanoparticles carrying HRPII, can induce brain inflammation and tissue damage (106, 107). These data indicate that EVs as well as non-EV particles hold molecular insights into vascular dysfunction.

### Severe malaria and EVs

Severe malaria (SM) is characterized by clinical or laboratory evidence of vital organ dysfunction, predominantly caused by *P. falciparum*, though recent findings indicate that *P. vivax* can also lead to severe disease (108). The defining characteristics of SM include impaired consciousness, acidosis, hypoglycemia, hyperparasitemia, severe anemia, renal impairment, jaundice, pulmonary edema, and bleeding from various sites (109).

Recent investigations by Thomas et al. showed the anti-inflammatory potential of EVs loaded with miR-451a and let-7i-5p during heme-induced inflammation (110). These EVs demonstrated the ability to attenuate heme-induced inflammation, correlate with malaria severity, and reduce *P. falciparum* counts. The study also highlighted the capacity of EVs to modulate inflammatory responses in human-induced pluripotent stem cell-derived endothelial cells (hiPSC-ECs) and influence the transcription of key inflammatory markers.

The impact of inflammatory extracellular vesicles (inf-EVs) during the blood stage of *P. berghei* infections has been also been explored (97). It was found that inf-EVs exacerbate liver injury, enhance proinflammatory responses, and trigger macrophage M1 polarization (97). Multiple microRNAs within inf-EVs were identified as potential mediators, targeting genes that inhibit M1 polarization in macrophages. These findings highlight the critical role of inf-EVs in liver injury and inflammation during *P. berghei* infections.

### EVs in cerebral malaria

The initial human investigations into MVs in CM identified increased concentrations of circulating endothelial cell-derived microparticles (MPs) and tumor necrosis factor (TNF) in Malawian patients during the acute phase of pediatric CM (44) (Fig. 3). Of interest, these heightened levels reverted to baseline after the infection subsided. Significantly, the substantial elevation of MPs was distinctive to the neurological complication, as it did not manifest in the cases of uncomplicated malaria or severe anemia.

A pivotal study by Valéry Combes and colleagues revealed the significance of MVs in CM. Mice with a deleted ABCA1 gene exhibited complete resistance to CM and this deletion led to a concentration reduction of MV and procoagulant activity, suggesting their involvement in CM pathogenesis (102). This study prompted further investigations that identified associations between ABCA1 gene promoter polymorphisms and plasma MV levels in malaria patients. The −477T and −320G polymorphisms, along with the T-G haplotype, were suggested to confer a protective effect against SM. Additionally, MV production was heightened in febrile patients during *P. falciparum* infection, with the −477C and −320G polymorphisms linked to increased MV production. The mutant genotype of the ABCA1 gene promoter was proposed to influence lower MV production during malarial infection, impacting the severity of malaria in humans (98).

In CM, three significant features are directly linked to MVs and exosomes: endothelial activation, endothelial adhesion for *Pf*-iRBCs, and the release of endothelial MVs (99, 101) (Fig. 3). Endothelial MVs produced after TNF activation have procoagulant and pro-inflammatory properties, similar to the cell of origin. MVs participate in the worsening of the endothelial lesion, by locally activating coagulation and potentiating leucocyte adhesion, suggesting a pathogenic role in the development of the cerebral syndrome (45).

In typical physiological circumstances, circulating MVs are derived from various cell types, especially from platelets, erythrocytes, leukocytes, and endothelial cells. However, the release of MVs can be indicative of cell activation and apoptosis (111). In addition, the elevated abundance of aminophospholipids on the MV surface provides binding sites for clotting factors such as IXa, VIII, Va, prothrombinase, and tenase and facilitates their involvement in cell-to-cell interactions, signaling, inflammation, coagulation, and vascular function (45) (Fig. 3).

Furthermore, platelet-derived MVs have the potential to serve as a valuable biomarker for monitoring patients with CM. This is supported by the higher levels, association with important clinical and biological factors, and normalization of the levels after remission (19). However, there is a discrepancy as another study revealed that erythrocyte-derived MVs are predominant, contrasting with lower platelet-derived MVs in CM patients (98). Nevertheless, it was observed that overall levels of circulating MVs derived from various cells were elevated in malaria patients, suggesting potential cell activation. The same study emphasized the importance of TNF and MV production in determining the severity of malaria (98). More recently, Pierre-Yves Mantel and his group demonstrated *in vitro* that human microglia can internalize EVs from malaria-iRBCs, leading to alterations in gene expression of the pro-inflammatory cytokine TNFα, and downregulation of the immune suppressive cytokine IL-10 (100).

In recent years, it has been observed that the intravenous administration of mast cell-derived EVs (MCs-Exo) resulted in a shortened survival time, aggravated brain histopathological damage, and increased the incidence of experimental CM (ECM) in PbANKA-infected C57BL/6 mice (112). It is suggested that MCs-Exo may exacerbate ECM pathogenesis, potentially by amplifying the host’s pro-inflammatory response, activating brain microvascular endothelial cells, and inducing blood-brain barrier (BBB) breakdown (112).

### Placental malaria and EVs

Placental MPs and microRNAs play a role in maternal tolerance towards the fetus and can also contribute to pregnancy pathologies such as pre-eclampsia (113). However, placental malaria was not associated with changes in MV levels but with changes in miRNA originating from the placenta and present in circulating MVs. For instance, miR-517c, belonging to the cluster C19MC, has been identified as an immunomodulatory factor both in pregnancy and tumorigenesis. The upregulation of miR-517c has been correlated with increased placental weight, pre-eclampsia, and recurrent spontaneous abortion (114) (Fig. 3). These findings suggest that placental miRNAs have the potential to serve as biomarkers for detecting placental malaria infection during gestation.

## EVs AS BIOMARKERS

The molecular cargo of EVs encompassing proteins, nucleic acids, glycoconjugates, and lipids, mirrors the physiological condition of their originating cells. Given their ubiquitous presence in bodily fluids, EVs are promising tools for biomarker discovery (13). Ideal biomarkers for diseases should be disease-specific and diminish post-treatment, ideally offering prognostic insights. Notably, parasitic infectious diseases may give rise to biomarkers from either host or pathogen sources (115, 116).

In malaria, particularly with *P. falciparum* and *P. vivax*, elevated levels of circulating EVs have been linked to clinical symptoms and disease severity, suggesting the potential for EV concentration as malaria severity biomarkers. One of the early pieces of evidence arises from a clinical study involving Malawian children, revealing elevated levels of EVs from endothelial cells. Specifically, patients with *P. falciparum* CM exhibited a sixfold increase in endothelial-derived EVs compared to controls (44). The same group, reported some years later another study in Cameroon investigating cell-specific MV patterns in severe malaria patients, particularly CM, aiming to understand vascular activation’s role in CM. The study showed that platelet-derived MVs were highly prevalent, and their concentrations exhibited a notable correlation with both the depth of coma and thrombocytopenia in CM patients (19).

Another field study conducted in Brazil demonstrated the indirect link between circulating platelet MVs and the severity of vivax infections (20). One year later, a field study conducted in Thailand examined red blood cell-derived MVs in *P. falciparum*, *P. vivax*, and *P. malariae* infections. This study validated elevated levels of MVs during infections and their indirect correlation with severity (45).

Recent proteomic studies utilizing liver-humanized mouse models capable of supporting *P. vivax* infections (117) have facilitated investigation into *Pv*EVs. Notably, parasite proteins were identified in EVs derived from plasma of these infected liver-humanized mouse models, opening new avenues for biomarker research in vivax liver infection, including hypnozoites (68). Moreover, a recent investigation exploring plasma EVs as malaria biomarkers unveiled significant variations in surface EV proteins between healthy individuals and malaria patients. CD106 emerged as the foremost discriminatory human marker (118).

In terms of nucleic acid composition, several investigations have demonstrated an increased release of EVs from different cell types, such as endothelial cells, RBCs, and platelets, harboring small RNA cargo, which aligns with the severity of malaria infection (46, 80). Recent research indicates differential expression of miR-451a and let-7i-5p in sickle cell disease (SCD) erythrocytes and *Plasmodium*-infected cells. Examining plasma exosomal miRNAs from various SCD genotypes (+/−malaria) revealed higher levels of let-7i-5p and miR-451a in HbSS- and lower levels in HbSS+ individuals compared to other genotypes (119). Altogether, these data indicate the potential of EVs during malaria infections as novel biomarkers; yet, differences in isolation methodologies, as well as lack of validation of some potential biomarkers, presently limit their value.

## EVs AND VACCINE DEVELOPMENT

Exosomes have been studied in the context of vaccination using the non-lethal murine model *Plasmodium yoelii* 17X-BALB/c, which mimics human malaria caused by *P*. *vivax* (120, 121). Thus, immunizing mice with exosomes isolated and purified from reticulocytes of experimental infections, elicited IgG antibodies against *P. yoelii*-iRBCs, increased reticulocytosis, and shifted cell tropism to reticulocytes of the lethal *P. yoelii* 17XL strain (32). Indeed, when combined with CpG oligodeoxynucleotides, exosome immunizations provided complete and long-lasting protection against lethal infections in approximately 85% of tested mice (32). Remarkably, this immune response activated effector memory T cells in the spleen, resulting in protection against initial and subsequent lethal *P. yoelii* infections (121). To advance this vaccination approach to *P. vivax* malaria, a recent analysis of reticulocyte-derived (R)EVs obtained from human patients revealed approximately 70 *Plasmodium* proteins, including immunogenic rhoptry proteins and MSP1 (68). These findings suggest that malaria-derived EVs can act as antigen-presenting entities, offering a promising avenue for vaccine development. Of importance, in another immunization study using EVs from the *P. yoelii* NL strain in combination with CpG, C57BL/6 J mice were barely protected from parasitemia; yet, the survival rate was increased and ameliorated changes in the brain, including a decrease in inflammatory changes and sequestration of parasites (122). Last, mice injected with EVs from *P. berghei*-infected mice, not only survived initial severe infection but also developed lasting immunological memory, providing immunity against subsequent infections (123). *Plasmodium* parasites use immunosuppressive EVs to evade host immune responses, impacting malaria vaccine development for long-term anti-parasite immune responses. This implies immune response-inducing EVs derived from infected reticulocytes may be more immunogenic and suitable for targeted immunogen administration.

Previous research has focused on using synthetic nanoparticles and microparticles for delivering parasite DNA (pDNA) or proteins, with PLGA being a prominent polymer. Shan Liu and colleagues utilized an ultrasonic atomization technique to produce PLGA microparticles loaded with concentrated malaria plasmid VR1020-MSP119, paving the way for large-scale production of clinically effective malaria pDNA vaccines (123, 124). The involvement of EVs in malaria suggests their potential as a vaccine delivery system, with PLGA-coated iRBC-derived EVs demonstrating immunization capability in mice. Controlled release of loaded antigens in biodegradable microparticles indicates prolonged functional antibody responses, enhancing the efficacy of anti-malaria vaccines (124).

## CONCLUDING REMARKS

Research on EVs in malaria has significantly increased since their first description as microparticles correlating with disease severity to their potential as novel intercellular communicators, biomarkers of disease, and therapeutic agents. Several studies have demonstrated that EVs play multifaceted roles in malaria pathogenesis and host-parasite interactions, influencing immune responses, gene expression, inflammatory responses, and disease severity; thus, emphasizing the need for more research into their mechanisms of action. However, there are major discrepancies in the molecular cargo of EVs obtained from *in vitro* culture as opposed to those obtained from patients, notwithstanding that most EV-mediated molecular insights come from such *in vitro* studies. These data indicate that rigor in standardizing methods for isolation and purification of EVs should be reinforced, to precisely associate the molecular cargo of EVs with pathophysiological functions induced by EVs as well as to distinguish them from non-EVs particles. Moreover, these data also indicate the need for transitioning to studies involving EVs obtained directly from patients to unveil the physiological role of EVs in natural human infections. In addition, key gaps in our knowledge about EVs (Box) remain to be unveiled for a comprehensive understanding of the molecular basis of pathophysiology and their use as novel diagnostic tools and therapeutic agents against malaria.

**ВОХ. Key gaps and perspectives in the research of EVs in malaria**

- The precise molecular mechanisms that govern EV release from *Plasmodium*-iRBCs are not fully understood, and the specific triggers and regulatory proteins involved in this process remain unclear.
- Although EVs released during malaria infection carry a variety of host and parasite molecules, the mechanisms that select these molecules for EV packaging are poorly characterized.
- EVs from malaria-infected cells obtained from clinical isolates as well as from *in vitro* culture indicate that these nanovesicles hold valuable insights into the pathophysiology of infections. Yet, validation of results in natural infections is imperative to avoid misleading guidance of alternative control efforts.
- High discrepancies between the protein cargo of EVs obtained from *in vitro* culture as opposed to those from patients underscore the need for more research using clinical samples to accurately extrapolate the EV composition and physiological role during natural infections.
- How the interaction between *Plasmodium* and host immune cells influences the quantity and content of EVs, and how this might contribute to immune evasion or disease progression, is an important research area that lacks comprehensive insights.
- Incomplete data availability across OMICs studies, differences in EV isolation methods, parasite life stages, selection criteria, and validation limit the value of the molecular cargo identified for biomarker discovery and vaccine development.
- The absence of rigorous standardization in current methodologies for the isolation and characterization of EVs in parasitic diseases, including malaria, is a major confounding factor in determining whether the observed effects are strictly related to EVs as opposed to non-EVs. Recent guidelines to standardize such methods have been published (35).

## SUPPLEMENTAL MATERIAL

Figure S1 mbio.02304-24-s0001.docx Life cycle of *Plasmodium* spp., highlighting new cryptic erythrocytic stages.

Supplemental legend mbio.02304-24-s0002.docx Legend for Fig. S1.

Table S1 mbio.02304-24-s0003.xlsx Details of samples and processing of EVs of the seven studies included in the meta-analysis.

Table S2 mbio.02304-24-s0004.xlsx List of *P. falciparum* proteins.

Table S3 mbio.02304-24-s0005.xlsx List of human proteins.

Table S4 mbio.02304-24-s0006.xlsx List of proteins shared in *in vitro* studies.

Table S5 mbio.02304-24-s0007.xlsx Shared proteins between *in vivo* and *in vitro* studies.

Table S6 mbio.02304-24-s0008.xlsx List of *P. vivax* proteins.

Table S7 mbio.02304-24-s0009.xlsx Compilation of effects reported in the literature.

ASM does not own the copyrights to Supplemental Material that may be linked to, or accessed through, an article. The authors have granted ASM a non-exclusive, world-wide license to publish the Supplemental Material files. Please contact the corresponding author directly for reuse.
