# Supplementary material for: Extracellular vesicles in malaria: proteomics insights, in vitro and in vivo studies indicate the need for transitioning to natural human infections
Source: mBio. 2025 Jan 27;16(3):e02304-24. doi: 10.1128/mbio.02304-24 (PMC11898581; doi:10.1128/mbio.02304-24)
Supplement: Figure S1 — Life cycle of Plasmodium spp., highlighting new cryptic erythrocytic stages. [file mbio.02304-24-s0001.docx]

**Supplementary Text for:**

**Multiple Sclerosis and Infection**

History, EBV, and the Search for Mechanism

Elliott D. SoRelle^1,*^ & Micah A. Luftig^1,*^

^1^Dept. of Molecular Genetics & Microbiology, Center for Virology, Duke University, Durham, NC, USA, 27710

^*^Corresponding authors (emails: [elliott.sorelle@duke.edu](mailto:elliott.sorelle@duke.edu) and [micah.luftig@duke.edu](mailto:micah.luftig@duke.edu))

**Supplementary Table of Contents**

| **Supplement: A Primer on Multiple Sclerosis** | |
| --- | --- |
| **History and Basics of Multiple Sclerosis** | 4 |
| Early Accounts | 4 |
| Formal Descriptions | 5 |
| Giving the Disease its Name | 7 |
| Definition, Diagnosis, and Prognosis | 7 |
| MS Disease Courses | 9 |
| Initial Neurologic Events – Clinically Isolated Syndrome and Optic Neuritis | 9 |
| Relapsing-Remitting MS | 10 |
| Primary and Secondary Progressive MS | 10 |
| Pediatric MS | 10 |
| Other MS Variants and Related Disorders | 11 |
| **MS Lesions** | 11 |
| Location and Type | 11 |
| Composition | 12 |
| What Kind of Disease is MS? | 12 |
| Peripheral Immune Cells | 13 |
| T cells | 13 |
| B cells | 13 |
| Monocyte-Derived Macrophages | 14 |
| Cells of the CNS | 14 |
| Neurons | 14 |
| Microglia | 15 |
| Astrocytes | 15 |
| Oligodendrocytes | 16 |
| Multicellular Complexity | 16 |
| **Evolution and Mechanisms of Disease-Modifying Therapy (DMT)** | 17 |
| Interferons | 18 |
| IFNγ | 18 |
| IFNβ | 18 |
| Synthetic Small Molecules | 19 |
| S1P Modulators | 19 |
| Fumarates | 19 |
| Glatiramer Acetate | 20 |
| Mitoxantrone | 20 |
| Cladribine | 21 |
| Antibodies | 21 |
| Anti-CD4 | 21 |
| Anti-VLA-4 | 21 |
| Anti-CD52 | 22 |
| Anti-CD20 | 22 |
| Treatment Summary and Perspective | 22 |
| **MS Epidemiology and Risk Factors** | 23 |
| Global and Regional Incidence | 23 |
| Age of Onset and Disability | 23 |
| MS Prevalence by Race and Ethnicity | 23 |
| Sex Bias in MS Susceptibility | 24 |
| Genetic Predisposition | 24 |
| Environmental Risks | 25 |
| Vitamin D | 25 |
| Smoking | 26 |
| Other Environmental and Lifestyle Factors | 27 |
| **Footnotes** | 28 |
| **References** | 29 |

**Supplement: A Primer on Multiple Sclerosis**

“…the end of all our exploring will be to arrive where we started and know the place for the first time.”

– T.S. Eliot, Little Gidding^1^

**History and Basics of Multiple Sclerosis**

Early Accounts

The earliest evidence for symptoms associated with multiple sclerosis (MS) lies in historical records describing individuals with neurologic impairment now recognized as characteristic of the disease. In hindsight, these accounts range from uncertain to clinically definite cases. For example, the saga of Iceland’s patron saint, Thorlak Thorhallsson (1133-1193), recounts the tale of Halldora, a young woman who became severely asthenic and bed-ridden^2^. Halldora was reported to have been “cured” sometime between 1193 and 1199, a miraculous outcome posthumously attributed to Bishop Thorlak. While such an outcome belies the medical reality of MS, it is conceivable that a temporary remission of Halldora’s symptoms may have been mistaken for a cure. A century later, another young Icelandic woman named Halla was reported to have experienced an acute episode of binocular blindness and impaired speech (dysarthria) that lasted for weeks but subsided. It is unclear whether Halla experienced further episodic symptoms or disease progression^3^. Perhaps the earliest compelling record of possible MS comes from the 14^th^ century in Schiedam, Holland. In late 1395, a fifteen-year-old girl named Lidwina suffered and recovered from an acute illness. Several months later, she fell while ice skating with friends and fractured several ribs; it is implied that the fall may have been related to muscle weakness in her lower body, as her ability to walk was thereafter substantially impaired. This marked the beginning of nearly four decades during which Lidwina suffered progressive neurologic symptoms with periods of remission. Among her many documented symptoms were worsening vision, paralysis, pain, trigeminal neuralgia, and difficulty swallowing^4^. Lidwina’s long and progressive course of neurodegeneration were reflected in Richard Gough’s early 18^th^ century account of Margaret Davis, a parishioner in Myddle, Shropshire, England. For more than twenty years leading up to her death in 1701, Margaret suffered increasing “paine and lameness in her limbs, and made use of several remedyes for curing therof, butt all proved ineffectual.”^4,5^. Notwithstanding the lack of formal diagnoses in clinical settings, many similar accounts throughout the historical record – particularly those assiduously curated by Murray^4^ – evoke the picture of a debilitating disease that has long been with humans. Among these documents, Augustus d’Esté’s personal diary is widely considered to be the first known definitive case of MS. The certainty in retrospective diagnosis of d’Esté’s illness – a relapsing and remitting condition followed by progressive disease – is owed to the meticulous notes he kept beginning at age 28 until his death at 54 (1822-1848)^4,6^. Though detailed in its description of symptoms and desperately sought remedies, d’Esté’s diary necessarily lacked insight into clinical features or the possible underlying cause. However, the contemporaneous work of several clinicians across Europe would soon provide essential early clues to the disease^7^.

Formal Descriptions

In 1824, Charles Prosper Ollivier d’Angers published a compendium of spinal cord pathologies based on gross examination. Within this work, Maladies de la moelle epiniére, d’Angers presented the first formal medical report – described as ‘myelitis’, possibly attributable to infection – that can be definitively identified as a case of relapsing-remitting MS. The patient, who had experienced “a period of feeling tired and languid at age 17”^4^ was a 20 year-old man who presented with limb weakness that would progressively worsen over the ensuing 30 years. In addition, d’Angers noted that the patient lost feeling in his legs in response to hot water treatment. However, there does not appear to be post-mortem follow-up that might have identified the underlying anatomical injury. Other early case reports have been reviewed in detail^4^, several of which deserve particular attention.

As a medical student at the University of Glasgow in the 1820s, Robert Carswell quickly became known for his talents in pathology and accurate medical illustration. Carswell was thus commissioned by a pre-eminent Scottish physician, Dr. John Thomson, to compile anatomical depictions of diverse human pathologies to accompany Thomson’s medical lectures. Between 1822 and 1831, this task led Carswell back and forth between Scotland and France’s public hospitals in Lyon and Paris, where regular anatomical lectures were easy to attend and ample cadaver tissues were available for study^4,8^. The product of Carswell’s efforts, published in parts and completed in 1838, was Pathological Anatomy: Illustrations of the Elementary Forms of Disease, a beautifully detailed collection of watercolors, drawings, and accompanying pathologic descriptions of diverse human maladies. Among renderings of various inflammatory diseases, mortification, and cancers is the first known clinical depiction of MS pathology. In Plate 4 of the section titled ‘Atrophy’, Carswell presented ‘a peculiar diseased state of the [spinal] chord and pons Varolii.’ Based on post mortem samples derived from two French patients, the illustration shows ‘remarkable lesion[s] of the spinal cord accompanied with atrophy’ **(Figure S1)**.

**
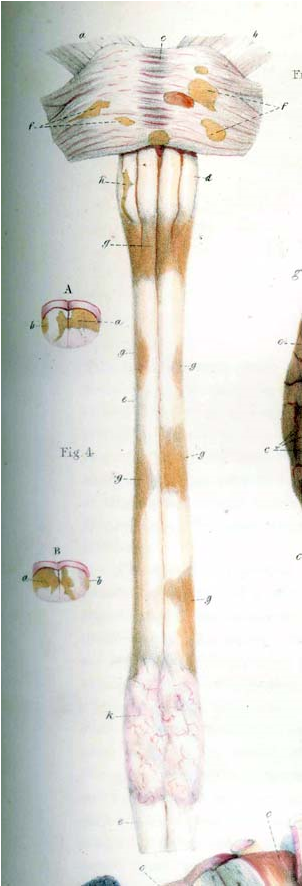
**
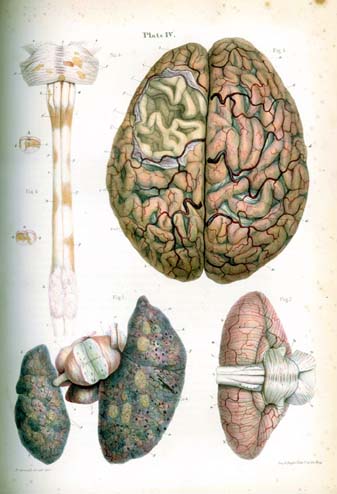


**Figure S1. Left, Plate 4 (“Atrophy”) of Robert Carswell’s *Pathological Anatomy* (1838).** The first known anatomical rendering of multiple sclerosis lesions of the spinal cord, illustrated by The Scottish medical student Robert Carswell^7^. **Right, Expanded view of spinal cord lesions from Plate 4.**

Despite the artistic accuracy, Carswell conceded that:

“…I could not ascertain that there was anything in the character of the paralysis or the history of the cases calculated to throw any light on the nature of the lesion found in the spinal cord.”^7^

At nearly the same time, the French pathologist Jean Cruveilhier published an installment of his physiological atlas, L’Anatomie pathologique du Corps Humain, that depicted similar disseminated lesions of the central nervous system (CNS)^9-11^. For this disease, Cruveilhier suggested the central pathologic importance of inflammation^10^. But as in Carswell’s effort, a link between lesion pathology and clinical symptoms as a distinct disease remained lacking in Cruveilhier’s analysis. In 1863, the German pathologist Eduard von Rindfleisch contributed an important refining observation for the disseminated CNS lesions documented by Carswell and Cruveilhier. In Histologisches Detail zu der grauen Degernation von Gehirn und Rückenmark (Histological Detail on the Gray Degeneration of the Brain and Spinal Cord)^12^, Rindfleisch described such lesions with particular attention to their vascularity:

“All vessels running inside the foci, but also those which traverse the immediately surrounding but still intact parenchyma are in a state characteristic of chronic inflammation.”^12^

Thus by 1863, medical literature across Europe contained anatomical renderings and pathologic descriptions suggestive of a distinct neurodegenerative and inflammatory disease that manifested at spatially disseminated sites^4,7,9,12^. Five years later, the French pathologist and neurologist Jean-Matin Charcot would formally link these lesions with clinical symptoms of neurologic dysfunction, thereby defining a new diagnosis for an old human disease.

Giving the Disease its Name

“To Dr. Charcot unquestionably belongs the credit of distinguishing this condition from other paralytic disorders…, of recognizing the pathological features, and tracing its clinical history.”^4,13^

Jean-Martin Charcot completed an internship in rheumatologic disease at Paris’s Hôpitaux Salpêtrière in 1853 and obtained his professorship in 1860. Despite his early work, Charcot’s landmark medical contributions advanced the study of neurologic diseases, which he came to via collaboration and mutual instruction with the neurologist and electrophysiologist Guillaume Benjamin Armand Duchenne^4^. Having developed an interest in cataloguing diseases of the nervous system, one of Charcot’s great clinical innovations was to systematically track groups of patients at Salpêtrière who presented with neurologic symptoms, up to and including post mortem tissue analyses. This process enabled Charcot to correlate symptoms with clinical pathology and classify distinct neurologic conditions with enhanced precision. Along with Edmé Vulpian, Charcot observed several cases of a condition characterized by a tremor distinct from that of Parkinson’s Disease (PD). Autopsies of patients who had suffered from this tremor (among other symptoms) consistently revealed the presence of what Carswell, Cruveilhier, and Rindfleisch had portrayed in their atlases of pathology: ‘la sclérose en plaques disseminée’ – disseminated sclerosis (replacement with connective or fibrotic tissue) of the brain and/or spinal cord. Charcot further described the presence of gliosis and adipose cells within the lesions and proposed the diagnostic symptom triad of intention tremor, nystagmus (involuntary repetitive eye movements), and dysarthria (scanning or impaired speech) to distinguish the condition from PD. So-named for the signature occurrence of island-like lesions present at various sites in the CNS, the diagnosis of disseminated sclerosis, or insular multiple sclerosis in English-speaking countries, was ultimately simplified to multiple sclerosis^4,14^.

Definition, Diagnosis, and Prognosis

Multiple sclerosis (MS) is clinically characterized by chronic neuroinflammatory demyelination, gliosis, and neurodegeneration^15^. An ever-increasing body of evidence indicates that inflammation in MS is mediated by autoimmune responses, and that unresolved inflammatory lesions precipitate chronic, multifocal demyelination and eventual axon loss within the CNS. While the general outcome of MS is increasing disability due to neurodegeneration, individual presentations and neurologic symptoms can vary widely depending on lesion burden and distribution. As Arnold Edwards wrote in 1895:

“In disseminated sclerosis we find a diffused and vague picture…it teases and weakens various parts of the central nervous system, so that we have a great number of symptoms mixed up together. Considering the irregularity of the sclerotic process, there will be, naturally, considerable diversity as regards the clinical signs in individual cases…”^16^

The precise course of MS varies substantially within and across individuals. Presentations can range from recurrent episodes of impaired vision, limb numbness, ataxia, or other symptoms lasting at least 24 hours (“attacks”) followed by periods of remission to progressively worsening disability. Notwithstanding, Charcot’s neurologic triad provided physicians with an initial, albeit imperfect, rubric for diagnosing the disease*.

Unsurprisingly, accepted MS diagnostic criteria have evolved substantially with clinical and technological advances since Charcot’s framing in 1868^17,18^. In 1965, Schumacher and colleagues formalized guidelines for MS as the diagnosis for people aged 10-50 years who experienced at least two clinical relapses and neurologic symptoms unexplained by differential diagnosis^19^. Poser and colleagues further delineated “Definite” from “Probable” MS diagnoses depending on the number of attacks experienced; the extent of clinical evidence for the disease; and paraclinical evidence of CNS lesions that do not produce active clinical signs but may have been responsible for previous neurologic symptoms (based on evoked response tests, CT or MR imaging, e.g.). Under the Poser Criteria, definite and probable MS cases could be further subdivided by evidence type into “Clinical” and “Laboratory-Supported” groups, wherein measurements of elevated intrathecal IgG or the presence of oligoclonal IgG bands (OCB) ^20^** – an indicator of local antibody production against a limited antigen repertoire – in the CSF but not serum distinguished lab-supported MS diagnosis^21^. Elevated levels of myelin basic protein (MBP) in the CSF, a byproduct of myelin sheath destruction, can also provide support for MS diagnosis^22^. The McDonald Criteria further emphasized the importance of evidence for lesions “disseminated in time and space” to enable exclusive diagnosis of MS^23^. Specifically, the development of new CNS lesions consistent with MS plaques on MR imaging (T2-weighted or gadolinium (Gd)-enhancing lesions) at least three months after initial clinical presentation was defined as a threshold to demonstrate dissemination in time. The occurrence of two or more attacks and detection of two or more lesions became sufficient to diagnose MS. The McDonald Criteria have been updated periodically over the past two decades to improve diagnostic accuracy and better delineate distinct disease trajectories^24-26^. Identification of additional CSF and especially serum biomarkers of inflammation (e.g., cytokines and chemokines) and neurodegeneration (e.g., neurofilament light chain (NfL)) remains a high priority, since such indicators may aid MS diagnosis, clinical course prediction, and response to therapy^27-31^.

There is currently no cure for MS. The disease inevitably progresses, and neurologic symptoms and cumulative disability become increasingly severe **(Figure S2)**. However, the nature and sequence of clinical events and symptoms can vary substantially. This natural variability has important implications for time to specific milestones in disease progression, quality of life and life expectancy, and treatment options for management^15^.

**
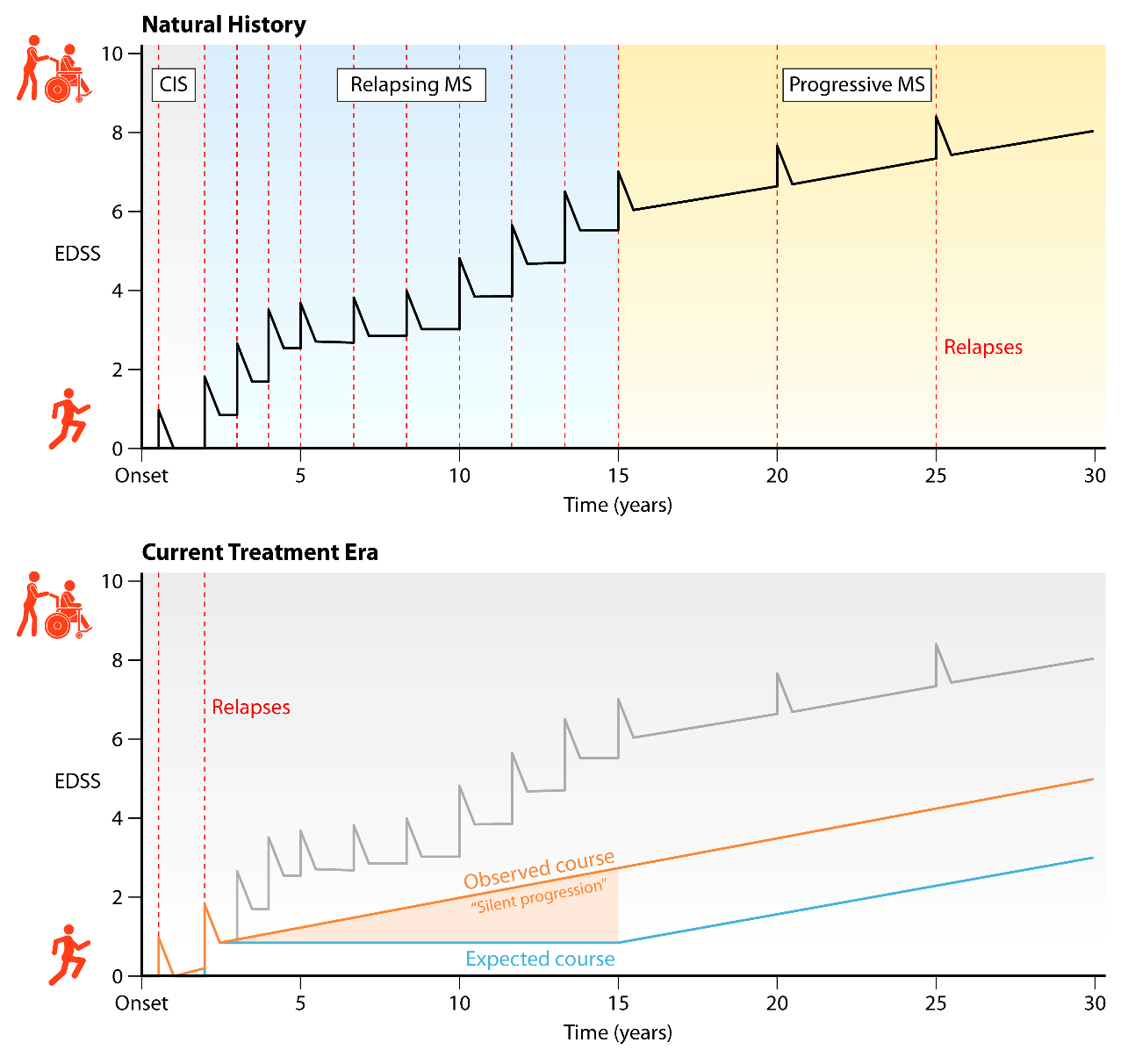
Figure S2. The evolving course of MS disease progression (adapted from ^15^ with permission from Elsevier).** The upper portion of the figure represents the natural history of relapse-onset MS before the introduction of effective treatments. In this era, disability progression during the relapsing phase was attributed to incomplete recovery from relapses, eventually giving way to relapse-independent disability, identified as SPMS. The lower portion illustrates the modern treatment era. With the widespread use of highly effective therapies, relapses are largely suppressed for most patients. However, a gradual progression of disability unrelated to relapses, referred to as "silent progression," has become apparent even during the relapsing phase. CIS = clinically isolated syndrome; EDSS = expanded disability status scale; SPMS = secondary progressive multiple sclerosis.

MS Disease Courses

“*Here is how things generally happen…the symptoms at first not very pronounced increase, sometimes in a progressive way, sometimes in fits and starts, and in a more or less rapid lapse of time the whole clinical aspect of multiple sclerosis is constituted.*”

– Pierre Marie

Les Maladies de la Moelle, 1892^32^

Initial Neurologic Events – Clinically Isolated Syndrome and Optic Neuritis

For roughly 85% of people who develop MS, disease onset manifests as an initial neurologic episode called Clinically Isolated Syndrome (CIS), with typical involvement of the optic nerves, spinal cord, or brain stem. CIS symptoms commonly include optic neuritis (ON) and blurred or otherwise impaired vision in addition to numbness, difficulty walking, fatigue, or loss of bladder control. T2-enhancing lesions may or may not occur in CIS, and lesions can be clinically silent if present^33^. Although lesions may not be detectable in CIS, MRI detection of higher lesion loads (that is, more lesions meeting the Barkhof-Tintoré Criteria) is prognostic for future neurologic episodes, higher scores on the extended disability status scale (EDSS)^34^ within five years, and greater likelihood of disease conversion to clinically definite MS (CDMS)^35,36^. About two thirds of people with CIS will experience additional symptoms and clinical progression within 15-20 years, and half of people with CIS will develop a progressive form of MS in the same interval^33,37^. For people with CIS who go on to develop CDMS, the median time to disease conversion is about four years^38^.

Relapsing-Remitting MS

The prototypical form of MS is a relapsing-remitting course (RRMS) that can be diagnosed based on recurrent episodes of neurologic dysfunction, an increase in T2-enhancing CNS lesions, leptomeningeal inflammation, and the presence of intrathecal OCB. 85% of people with RRMS experience disease flares at least annually, with neurologic episodes followed by periods of recovery^39^. Relapses generally indicate the formation of new lesions; while not strongly correlated with contemporaneous disability, higher lesion burdens are predictive of greater disability long-term^40,41^. Within 15 years of RRMS diagnosis, 80% of patients will experience some permanent functional impairment. And, just as diagnosis with CIS carries a high likelihood for conversion to clinically definite RRMS, a diagnosis of RRMS increases the probability of conversion to progressive MS. There is a roughly linear relationship between time and the risk for such conversion, with a 2.5% chance per year ^39^.

Primary and Secondary Progressive MS

MS initially presents as a progressive neurodegenerative disease in 15% of people who are diagnosed^39^. This primary progressive form (PPMS) presents later in life, at a median age of 40 versus 30 for RRMS, but has a poorer prognosis^42,43^. The first symptom of PPMS is often spastic paraplegia due to spinal cord atrophy. PPMS lesions are typically smaller, but the extent of axonal damage and microglial activation is greater than in RRMS^44^. However, RRMS also invariably converts to a progressive disease, termed secondary progressive MS (SPMS), typically within 10-15 years without disease-modifying therapy (DMT). PPMS and SPMS share many of the same basic clinical features. For example, each form can include periods with or without advancing disability (progression) that may or may not coincide with symptomatic relapses and new lesion formation (disease activity). Timing is therefore the predominant distinction between PPMS and SPMS^42,43^.

Pediatric MS

As early as 1902, it was known that MS rarely occurs in children^45^. Pediatric cases (onset before age 16) account for about 4% of MS diagnoses^46^. Lesions in the cerebellum and brainstem are common at pediatric MS onset, and most patients experience a relapsing-remitting course with frequent attacks and rapid recovery^47,48^. These patients consequently reach disability milestones (e.g., elevated EDSS scores) earlier in life, and half convert to progressive disease (SPMS) within 23 years of onset^46^.

Other MS Variants and Related Disorders

Several similar inflammatory neurologic conditions are variants of MS or closely related diseases^49^. Baló’s concentric sclerosis (encephalitis periaxialis concentrica), named for its distinctive patterning of white matter lesions, is a rare monophasic disease that is thought to be distinct from MS and does not require ongoing treatment^50^. Schilder’s disease (diffuse myelinoclastic sclerosis) is a rare form of pediatric MS characterized by especially large lesions within myelin-sheathed axon tracts called white matter (white matter lesions; WML) and life expectancy less than ten years^51^. Large sclerotic plaques that resemble neoplasms are characteristic of tumefactive MS, which has distinct early symptoms but eventually transitions to the RRMS course^52,53^. The tumefactive MS variant described by Otto Marburg in 1906 is a rare but aggressive disease that is now recognized to be frequently monophasic and may not require continuous treatment^54^.

Acute disseminated encephalomyelitis (ADEM) is another related neuroinflammatory disease that is often triggered by infection. While ADEM is not considered to be a variant of MS, children and adults diagnosed with ADEM are at elevated risk for developing MS^49,55^. Neuromyelitis optica (NMO)^56,57^ is another multifocal demyelinating disease that is distinct from (and rarer than) MS, although optic neuritis (ON) and myelitis are common symptoms in both. Unlike permanent vision loss in NMO, ON symptoms in MS are generally monocular and reversible^49^.

**MS Lesions**

Location and Type

Blood-brain barrier (BBB) breakdown and acute inflammation are early events in MS lesion formation^15^. These lesions, or plaques, occur in the brain and spinal cord as a result of CNS-infiltrating immune cells^53^. Many people with MS exhibit cord lesions, and joint consideration of brain and cord lesion burdens led to more patients meeting the McDonald criteria for plaque dissemination in space during early disease stages^58^. Individual lesions can exhibit one of several morphologies that typically correlate to distinct disease phases^15^ **(Figure S3)**. WML within perivascular spaces are the classical presentation, but lesions can also form in the leptomeninges (the arachnoid and pia mater) and cortex (gray matter lesions, GML). Focal WML are common in patients with RRMS, whereas GML and diffuse injury of normal-appearing white matter (NAWM) are characteristic of progressive stages of MS. Lesions can be further delineated as active or inactive depending on whether they contain cells mediating an ongoing inflammatory response^44^. Notably, chronically active (‘smoldering’) WML are significantly associated with disability progression^59,60^.


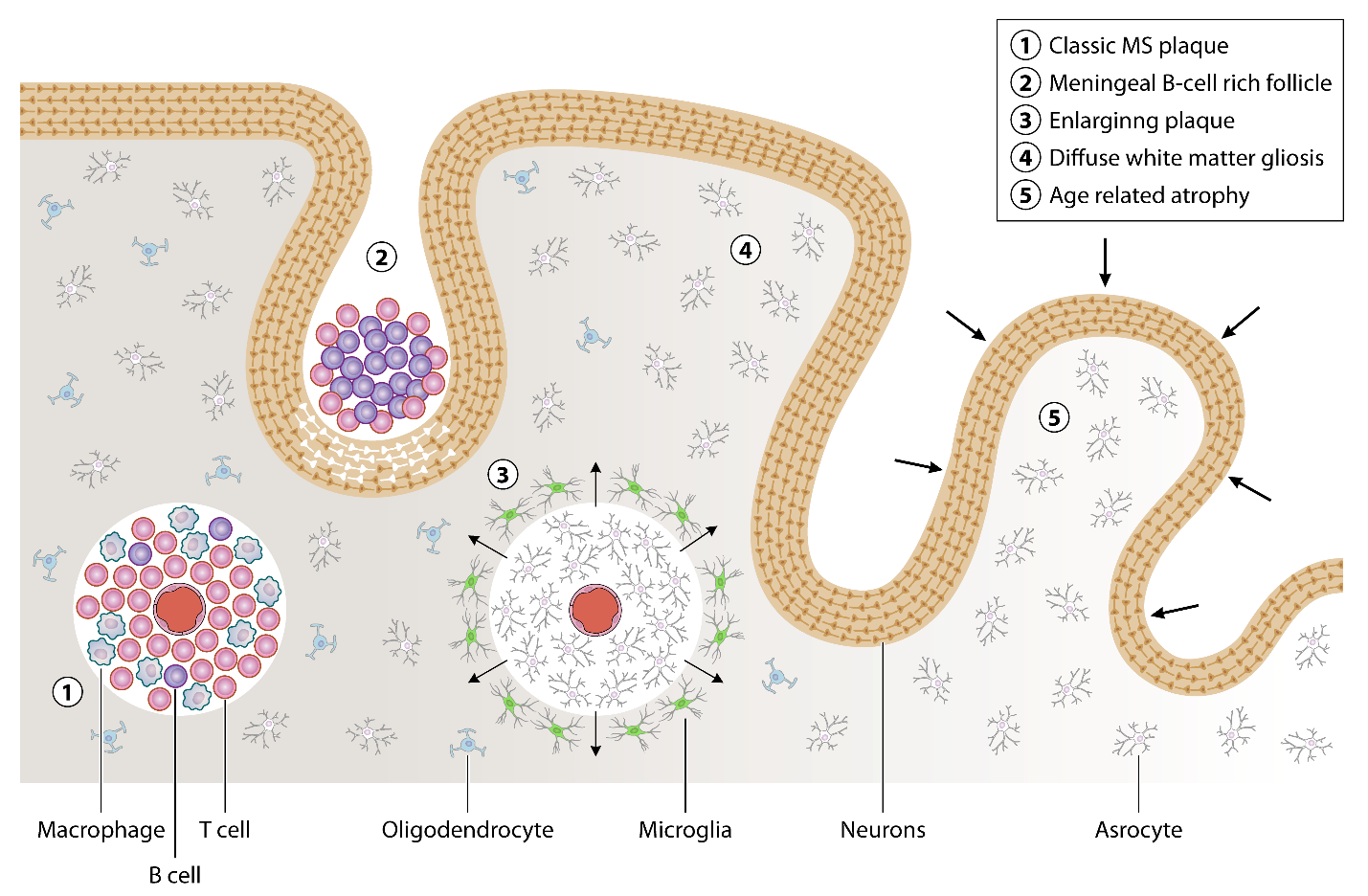


**Figure S3. Key pathological processes contributing to progressive multiple sclerosis (MS) (adapted from ^15^ with permission from Elsevier). (1)** Classic inflammatory white matter plaques are often linked to relapses but can also appear in patients with gradual progression without acute attacks. These plaques feature perivenous inflammation primarily involving lymphocytes and macrophages (visible on MRI through gadolinium enhancement indicating blood–brain barrier disruption), demyelination with activated microglia, sharply defined borders with glial scarring, and axonal loss accompanied by retrograde and anterograde degeneration. **(2)** B-cell–rich lymphoid aggregates located in the meninges, frequently in deep sulci, lead to cortical demyelination and neuronal loss in the underlying tissue. **(3)** Slowly expanding lesions arise from the gradual concentric growth of chronic plaques, marked by a rim of activated microglia at the edge, astrocytosis, stressed oligodendrocytes, and ongoing axonal injury. **(4)** Diffuse microglial inflammation and astrogliosis are widespread throughout CNS white matter, associated with reduced myelin density and continuous axonal damage. **(5)** Age-related neurodegeneration. CNS = central nervous system; MRI = magnetic resonance imaging.

Composition

Pathologically distinct lesions are heterogeneously composed of glia and neurons as well as infiltrating T cells, B cells, and macrophages. Proportions of immune subsets vary with lesion location – for example, leptomeningeal infiltrates resemble tertiary lymphoid structures with higher B cell frequencies than in conventional WML, which are instead predominated by T cells^53,61^. Increased meningeal infiltration by B and T cells is associated with greater subpial WML size and demyelinating activity^62^. Antibody-secreting cells (ASCs) also preferentially accumulate in lesions relative to surrounding normal tissue, especially in newer plaques^63^. By contrast, cortical GML (historically under-detected by MRI) contain fewer CD3^+^ lymphocytes and CD68^+^ microglia and macrophages but exhibit substantial demyelination, neuronal apoptosis, and neurite transection^64,65^. The active lesion is a highly inflammatory milieu of activated innate and adaptive cells and local concentrations of complement, inflammatory cytokines, IgG, and immune chemoattractants (e.g., CCL family chemokines)^66-68^. Advances in single-cell and spatial biology techniques have generated high-resolution perspectives of previously unknown biological activities within well-known features such as the activated microglial rims that surround hypocellular cores in chronic active WML^69^.

What Kind of Disease is MS?

MS includes both autoimmune and neurodegenerative characteristics. Episodic attacks primarily involve immune-mediated inflammatory demyelination, and the progressive phase is generally associated with extensive neurodegeneration. However, neuronal atrophy can begin early in disease course and, conversely, chronic autoimmune inflammatory responses can persist late into clinical progression. Thus, MS may best be understood as the complex interplay of neuroinflammatory and neurodegenerative processes over time^39,70-73^. The nuanced pathology of the disease is further underscored by the observation that the initial site of CNS attack is not necessarily the origin for progressive disease^74^. Below, we consider autoimmune, neuroinflammatory, and neurodegenerative aspects of MS from a cellular basis – saving an expanded discussion of B cell-mediated pathogenesis for the main text.

Peripheral Immune Cells

Immune infiltration of the CNS is a critical event in MS pathogenesis. Activated autoreactive lymphoid cells that recognize CNS-expressed antigens induce cell- and antibody-mediated neuroinflammatory demyelination. Both infiltrating lymphoid and myeloid subsets respond to and potentiate these responses and their resolution.

T cells: Early studies of lesion composition implicated a pathogenic role for CD4^+^ T cells^75^. The discovery that MBP-reactive T cells drive experimental autoimmune encephalitis (EAE) in animals, a useful in vivo model approximating MS^76^, strongly supported this model. MBP-reactive CD4^+^ T cells are present in people with MS as well as healthy individuals, however these cells are significantly enriched in MS CSF and are more sensitive to IL2-induced activation^77^. Enriched CD80^+^ and CD86^+^ cells and high IL-12 concentration within lesions create a permissive environment for T cell activation and skewing toward Th1-like inflammation^78^. IL-12-mediated Th1 polarization of CD4^+^ T cells from MS patients in vitro leads to increased interferon gamma (IFNγ) production and reduced TIM3 expression versus CD4^+^ T cells from healthy controls^79^. Peripheral lymphocytes from people with MS contain higher frequencies of IFNγ-producing T cells than people with other neurologic conditions, and the elevated reactivity of T cells in the CSF toward MBP and other CNS autoantigens is consistent with T cell-mediated demyelination^80^. Further, T cells in MS lesions express CXCR3, the receptor for the inflammatory chemokines CXCL9 (MIG) and CXCL10 (IP-10) as well as CCR5, the receptor for CCL5 (RANTES); CXCL9, CXCL10, and CCL5 are all notably elevated in the CSF during MS relapses^81^. These data supported the hypotheses that MS pathology was predicated on the failed elimination of CNS-autoreactive T cell subsets^82,83^. Autoantigen-directed T cell responses in MS also implicate the importance of antigen processing and presentation by any number of antigen-presenting cells.^84^ Additional studies have revealed extensive and complex T cell-mediated cytokine production within MS lesions^85^.

B cells: CD19^+^ B cell and B cell-derived IgG^+^ ASC frequencies within MS lesions vary by location and disease stage^53,63^. Early evidence for B cell pathogenesis in MS stemmed from the discovery of myelin-reactive autoantibodies significantly associated with demyelination^86^. Subsequently, OCB and intrathecal enrichment of proliferating centroblast-like CD19^+^CD38^+^ B cells and CD80^+^CD86^+^ memory B cells were identified in MS CSF but not peripheral blood^87^. Thus, CNS-restricted B cell differentiation is commonly observed in MS neuroinflammation. However, B cell subsets also modulate antibody-independent neuroinflammation in the CNS (including meningeal follicles and parenchymal plaques) through production of cytokines including TNFα, lymphotoxin alpha (LTα), IL-6, GM-CSF, and IL-10^88-90^. Intriguingly, B cell-derived IgA^+^ effectors that originate within the gut mucosa can enter the CNS and secrete IL-10 to attenuate neuroinflammation^91^. B cell-mediated immunomodulation, even specifically within the CNS, is therefore highly context and niche-dependent.

Monocyte-Derived Macrophages: Peripheral monocyte-derived macrophages frequently co-localize with transected axons in MS lesions^92^, suggesting a pathologic association. Like T cells, CD14^+^ macrophages in MS CNS tissues express surface receptors (e.g., CCR5) that are responsive to inflammatory chemokines^93^. Peripheral monocytes from treatment-naïve MS patients also secrete elevated levels of pro-inflammatory cytokines (IL-6, IL-12) and upregulate co-stimulatory molecules (CD80, CD86) relative to those from healthy controls^94,95^. However, monocyte-derived macrophages may also mediate neuroprotective responses by clearing myelin debris and adopting anti-inflammatory states^96^.

Cells of the CNS

Irreversible neuron destruction underlies progressive, permanent disability in MS. This damage is mediated not only by infiltrating immune cells but also interactions among glia (microglia, astrocytes, and oligodendrocytes), which constitute 90% of cells in the brain^97^. Although precise pathogenic mechanisms vary by lesion, meningeal inflammation from tertiary lymphoid structures and smoldering inflammatory responses of CNS-resident immune cells each contribute to chronic neurodegeneration^98,99^.

Neurons: Neurons are the primary targets of MS neuroinflammatory responses. The individual neuron is coarsely composed of a cell body, dendrites, and an axon. Networks of neurons formed via interneuron synapses between axonal and dendritic termini enable electrochemical signal transmission to coordinate complex physiological functions and information storage. The efficiency and directionality of such transmission along subcortical axon tracts (white matter) critically depends on protective protein and lipid-rich myelin sheaths. Myelin-directed antibody- and cell-mediated immune responses degrade axonal insulation, leading to defective neurotransmission. Direct damage to axons abrogates interneuron signaling altogether. Consequently, axon loss in the neurodegenerative phase of MS is characterized by a significant reduction of brain volume^53^.

Microglia: Microglia are specialized CNS-resident macrophages derived from the early erythromyeloid lineage and represent around 12% of brain cells. Microglia can activate adaptive and innate responses to CNS insults^100^ and are present near WML^101^. Whether microglia mediate neuroprotective or neurodegenerative processes depends on the balance of their sensing, housekeeping, and defense functions, each of which can be markedly perturbed in neuroinflammatory conditions^102^. Microglia constitutively express toll-like receptors (TLRs) that, when stimulated by exogenous nucleic acids and other pathogen-associated molecular patterns (PAMPs), contribute to cellular activation and innate immunity^103,104^. Microglial activation involves a transition from ramified to amoeboid morphology; antigen presentation by major histocompatibility complex (MHC) class II molecules; upregulation of immune costimulatory molecules (e.g., CD40, ICAM1); secretion of inflammatory cytokines (IL-1β, IL-6, IL-18, IL-12, IFNγ, and TNFα) and immune chemoattractants including CXCL10 (IP-10); and generation of reactive oxygen species (ROS)^102-107^. Activated microglia thus promote paracrine recruitment of CXCR3^+^ immune cells to the CNS and, in the case of T cells, skew their profiles toward Th1-like inflammation^105,108^. ROS production by activated microglia is associated with pathology, since oxidative damage is typical in MS lesions – elevated ROS levels are associated with demyelination and can induce proinflammatory macrophage responses^100,107,109,110^.

Conversely, microglia indirectly confer neuroprotection by inducing oligodendrocyte precursor (ODP) differentiation into myelin-secreting mature oligodendrocytes (ODCs)^111^. Microglia also clear myelin debris in EAE models, although the potential benefit or detriment of this function could be contingent on subsequent presentation of myelin-derived antigens to T cells^112,113^. Notably, macrophages derived from infiltrating peripheral monocytes but not those that originate from activated microglia may induce demyelination^112^. Given this distinction, particular care must be taken to properly define macrophages lineages within MS lesions^114-116^.

Astrocytes: Astrocytes maintain neuronal synapses, regulate neurotransmitter and metabolite concentrations, and provide structural integrity to the BBB via interactions with brain vascular endothelial cells (BVEC)^117^. While autoantibodies reactive against astrocyte-expressed aquaporin-4 (AQP4) likely contribute to BBB deterioration and immune infiltration in NMO^118^, astrocytes have historically been considered as marginal players in MS pathology^119^. In a rat EAE model, IL-1β induces astrocytic VEGF-A production, which increases BBB permeability by downregulating tight junction proteins^120,121^. Activated astrocytes in chronic active MS lesions also secrete the immune chemoattractants CXCL10 and CCL2, which promote infiltration of CXCR3^+^CCR2^+^ macrophages^122^. Astrocyte-derived IL-6 and BAFF can enhance inflammation and predispose infiltrating B cells to survival and differentiation^123-125^; B cell survival and activation are also supported by astrocyte-secreted factors independent of IL-6 and BAFF^126^. Astrocytic VCAM-1 and ICAM-1 expression may facilitate extravasation of immune cells expressing VCAM-1 binding partner VLA-4 and ICAM1 ligands (LFA-1, ITGB2, ICAM-1)^127,128^. Activated astrocytes – possibly in response to TLR and/or complement receptor stimulation^125^ – may thus play important roles in BBB breakdown and immune cell recruitment to the CNS during early MS pathobiology.

Oligodendrocytes: Mature ODCs help protect and maintain axons by producing myelin. Cellular injury to ODCs via uncontrolled inflammation or oxidative stress impair this function, leading to remyelination failure and subsequent permanent damage. While axonal injury can occur in the absence of demyelination in MS^44^, the importance of ODC-mediated neuroprotection is apparent from the concomitant loss of ODCs, myelin, and axon integrity in progressive MS^129^. Specifically, ROS-mediated DNA damage within WML – possibly induced by activated microglia in the context of persistent inflammatory responses to innate stimuli^106^ – is associated with ODC loss^130^. Bystander damage to ODCs appears to contribute to neurodegeneration and is likely a consequence of immune defense activation and inflammatory responses.

Multicellular Complexity

Autoimmune and neurodegenerative processes mediated by individual cell types in MS become more complex in gestalt. This complexity poses a challenge to accurately defining sequential causes and consequences in the earliest stages of disease. As one illustration, nitric oxide (NO)-mediated cellular damage can be induced in neuron and glia co-cultures upon treatment with the inflammatory cytokines TNFα and IL-1β^131^. This result suggests activated glial pathogenicity in response to existing local inflammation, and microglia are known to produce TNFα and IL-1β when activated by innate stimuli such as lipopolysaccharide (LPS). However, astrocytes also produce NO under similar inflammatory conditions and secrete TNFα upon treatment with LPS or IFNγ^132-134^. Moreover, inflamed microglia prime astrocyte-mediated neurotoxicity via TNFα, IL-1α, and complement C1q leading to neuron and ODC death^135^. Thus, in physiologic context, glia-mediated neurodegeneration may be instigated upon detection of pathogenic species within the CNS and precipitate both local inflammation and recruitment of immune cells from the periphery. Alternatively, events in the periphery that contribute to immune recruitment to the CNS may drive neuroinflammation and indirectly promote demyelination and neuronal death via glial activation.

It remains to be definitively resolved which of these event sequences manifest during MS onset. In either case, auto- or cross-reactive cellular and humoral adaptive responses of infiltrating immune subsets also mediate neurodegeneration (discussed subsequently). Regardless of their precise origins, unresolved immune responses against persistent stimuli sustain and augment MS neurodegeneration. This is evident during later stages of disease within chronic active lesions, which are characterized by scarred hypocellular cores and active immune rims. In addition to glial cells and immune infiltrates, chronic active lesion edges contain neurons and ODCs that display upregulated stress signatures^69,122^. Notably, a pathologic axis of infiltrating lymphocytes, C1q-producing activated microglia, and reactive astrocytes has been confirmed within chronic active lesion rims at high resolution^136^. B cells in particular induce microglial pro-inflammatory cytokine responses (IL-6, IL-12, and TNFα) in vitro, which in turn potentiate B cell activation^137^.

The intricate interplay between immune dysregulation and neurodegeneration in MS depends on not only many cell types but also their variability in time and space. However, detailed spatiotemporal studies that capture the clinicopathologic diversity of the disease have several practical limitations. For example, comprehensive studies of chronic active lesions from the progressive disease phase are more feasible than those focusing on earlier lesions due to the nature of available post mortem samples. Similar genome-wide insights from early WML and leptomeningeal lesions – ideally in spatial context – are greatly needed to better understand disease onset, episodic relapse, and periods of remission. When possible, in situ examination of early cellular dynamics and their modification by gold-standard therapies will be invaluable for improving MS treatments and long-term outcomes.

**Evolution and Mechanisms of Disease-Modifying Therapy (DMT)**

Disease-modifying therapies (DMT) can significantly extend time to disease milestones and life expectancy for people with MS relative to the natural disease course. Recommended regimens vary by stage and continue to evolve with the development of new biologic strategies^15^. The most effective DMTs have immunomodulatory mechanisms of action, consistent with the centrality of perturbed immune homeostasis to MS pathology^138^. The history of MS therapeutics includes several surprises and counterintuitive results that have helped refine best clinical practices and our understanding of the disease.

Interferons

Interferons were tested in some of the first clinical trials for MS disease management. The divergent patient outcomes observed between IFNγ and IFNβ treatment have been highly instructive for improving immunomodulatory DMTs and defining underlying immune-mediated pathology in MS.

IFNγ

An early clinical trial tested IFNγ in patients with RRMS^139^ on the basis of early hypotheses of viral involvement in MS^140^, IFNγ-upregulated MHC class II antigen presentation^141^, and attenuated IFNγ production by MS PBMCs upon viral exposure^142,143^. While treatment yielded dose-dependent increases in HLA-DR^+^ circulating monocytes, 7 of 18 recipients experienced clinical exacerbations that implicated IFNγ in MS activity^139^. Subsequent studies found that elevated IFNγ precedes MS onset and relapses^144,145^ and IFNγ worsens demyelination in murine EAE^146^. While unsuccessful, these results offered vital clues to the adverse nature of enhanced antigen presentation and immune response activation.

IFNβ

By contrast, IFNβ trials for people with RRMS were clinically and radiologically promising and led to the first clinically approved DMT for MS. Intramuscular IFNβ injections significantly reduced new lesion formation and relapse rates in a dose-dependent manner. Treatment did not significantly alter disability scores (EDSS) between placebo and treatment arms in the original study^147,148^, though a subsequent trial found that IFNβ slowed disability progression^149^. IFNβ limits leukocyte access to the CNS, immune cell antigen presentation capacities, and potentiates a shift from pro-inflammatory (IFNγ, TNFα) to anti-inflammatory (e.g., IL-10, TGFβ) cytokine milieus^138,150,151^. Specific effects of IFNβ treatment for MS include preservation of BBB integrity^152^; attenuated T cell activation, chemotaxis, and inflammatory cytokine secretion^153^; fewer activated CD80^+^ B cells^154^; and reduced MHC class II expression by both B cells and glia^155^. These effects strongly implicate pathogenic B cell-T cell interactions and co-stimulation as well as ICAM-1^+^ / VLA-4^+^ (ITGA4) T cell extravasation facilitated by inflammatory damage to BVECs. Notably, IFNβ is not effective for treating PPMS^42^, nor is it the current standard of care for RRMS.

Synthetic Small Molecules

Many pharmacologic agents are available for effective treatment of MS in one or more stages. We highlight several that display clinical efficacy through individual or pleiotropic effects across immune cell populations. These underscore critical roles of peripheral immune cell activated and regulatory states, trafficking, and proliferation in MS neuroinflammation and disability progression. Treatment effects on B cell and CD8^+^ T cell functions are especially noteworthy.

S1P Modulators

B cell-T cell costimulatory interactions are essential for efficient generation of differentiated immune effectors within germinal centers (GCs)^156^. These responses also depend on effector lymphocyte egress from GCs into peripheral circulation. This exit depends on the sphingosine-1-phosphage (S1P) receptor S1PR1 and can be disrupted by the small molecule fingolimod (FTY720)^157^. Upon phosphorylation by Sphingosine Kinase 2 (SPHK2), fingolimod is converted to a S1P structural analog that antagonizes S1PR1 by inducing its internalization, phenocopying S1PR1 genetic deletion^158-161^. Thus, fingolimod desensitizes B and T cells to S1P and blocks their entry into circulation^162^. Fingolimod also promotes remyelination in experimental models via S1PR1-, S1PR3-, and S1PR5-dependent effects on microglial and astrocytic functions^163-165^. In clinical trials for RRMS, orally administered fingolimod significantly reduced relapse rate, new MRI-detected lesions, and disability progression versus IFNβ^166,167^. As for IFNβ, fingolimod did not significantly alter disability progression in a PPMS cohort^168^. Additional modulators of S1P family receptors including ozanimod, sipinomod, and ponesimod are also used for MS management.

Fumarates

Dimethyl fumarate (DMF) is metabolized in vivo to monomethylfumarate and subsequently to fumarate, a key metabolite of the tricarboxylic acid (TCA) cycle^169^. Originally approved as an anti-inflammatory treatment for psoriasis, DMF and its bioactive derivatives shift lymphocytes and microglia toward anti-inflammatory Th2 profiles^170^ and promote NRF2-mediated resistance to oxidative stress^171^, both of which can confer cytoprotection. In MS clinical trials, orally delivered DMF (BG-12) was well-tolerated and significantly reduced new Gd-enhancing lesions, relapse rate, and disability progression in RRMS^172-175^. While DMF significantly depletes all CD3^+^ T cells, reduction of CD8^+^ populations is significantly greater than that observed for CD4^+^ cells^176^. In the context of support for CD8^+^ cell CNS invasion and pathogenesis^177,178^, preferential cytotoxic T cell (CTL) depletion appears to contribute to DMF’s therapeutic value. DMF is currently one of several recommended treatment options for people with CIS and RRMS^15^. Other clinically approved drugs within the fumarate class include diroximel fumarate and monomethyl fumarate.

Glatiramer Acetate

Glatiramer acetate (GA; copolymer-1) is a synthetic biopolymer consisting of randomly ordered amino acids (Glu, Lys, Ala, and Tyr) present at high frequencies within myelin basic protein (MBP). Ironically, GA was the product of an effort to develop an EAE model induction method on the premise that it would mimic MBP-derived antigens. Contrary to that expectation, GA treatment conferred resistance to EAE induction^179^, likely by competing with native myelin-derived antigens for MHC binding to MBP-specific T cell activation and impairing microglia-T cell interactions^180-182^. GA also induces a shift from pro- to anti-inflammatory T cell responses as evident from increased IL-10 and decreased TNFα after treatment^183,184^. GA-induced regulatory responses of MHC-restricted CD8^+^ T cells mediate cytotoxic responses against GA-specific CD4^+^ T cells^185,186^. In clinical trials, GA was well-tolerated and effective at reducing relapse rate, disability progression, and delaying the conversion from CIS to clinically definite MS^183,187^, making it a viable treatment for early stages of disease (e.g., CIS) but not progressive MS^42,188^.

Mitoxantrone

Mitoxantrone is a doxorubicin structural analog originally developed to treat acute myeloid leukemia (AML). As a DNA intercalating agent, its primary mechanism of action in MS is to block T cell, B cell, and macrophage proliferation by disrupting DNA replication and repair. Consequently, mitoxantrone treatment results in impaired antigen presentation capabilities, attenuated inflammatory cytokine secretion, and reduced myelin destruction^189^. B cell-specific mitoxantrone effects are especially noteworthy: depletion of CD19^+^CD27^+^ memory B cells, reduced lymphotoxin and TNFα production^190^, and curtailed humoral responses due to induced apoptosis^191,192^. Although mitoxantrone is one of the comparatively few therapeutic agents with demonstrated efficacy for relapsing and progressive MS courses^193^, its cytotoxicity profile limits its use in clinic^15^.

Cladribine

Cladribine is another small molecule originally developed for cancer therapy (primarily B cell lymphomas) that has been repurposed to treat MS. Cladribine is an adenosine structural analog that, when triphosphorylated, preferentially accumulates in lymphocytes and may inhibit their proliferation by inducing irreparable DNA strand breaks^194^. Cladribine reduced MS relapse rates, disability, and lesion loads in a clinical trial^195^, likely due to B cell depletion^196^.

Antibodies

The MS therapeutic landscape has broadened to include effective monoclonal antibodies that deplete specific immune cell populations. Although early trials of CD4^+^ T cell depletion were unsuccessful, biologics that target CD8^+^ T cells and especially B cells have become standard DMTs for relapsing and progressive MS courses.

Anti-CD4

The classical view of MS as a T cell-mediated disease raised the prospect of targeted T cell depletion for therapeutic benefit. However, a phase II trial of the chimeric monoclonal anti-CD4 antibody cM-T412 (Priliximab) found negligible benefit to CD4^+^ T cell depletion based on radiologic primary endpoints^197^. CD4-targeted therapy notably had no effect on Th1-polarized cell frequencies and IFNγ production^198^.

Anti-VLA-4

While CD4^+^ T cell depletion was not therapeutically effective, inhibition of T cell entry into the CNS with anti-VLA-4 monoclonal antibody (natalizumab, Tysabri) successfully reduces relapse rate and cumulative disability in RRMS^199^. This benefit is derived from blocking the interaction between the T cell-expressed adhesion receptor VLA-4 (α4 integrin) and its cognate receptor, VCAM-1, expressed on BVECs^200,201^. Like IFNβ, anti-VLA-4 treatment also impairs T cell engagement with VCAM-1-expressing microglia to limit microglial inflammatory responses^202^. Despite these favorable effects, natalizumab triggered serious adverse events related to reactivation of John Cunningham polyomavirus (JC virus) in several patients during clinical trials (discussed in Part I). Thus, natalizumab is a therapeutic option reserved solely for people with MS who are seronegative or have a low titer of JC virus.

Anti-CD52

Pan-lymphocyte depletion with anti-CD52 (alemtuzumab) achieved delayed disability in people with RRMS but frequently led to adverse autoimmune events in a phase II trial^203^. In a phase III trial, alemtuzumab more effectively reduced MS relapses versus IFNβ but had no discernible advantage with respect to delaying disability progression^204^. Several studies suggest that reduced T cell frequencies are sustained with anti-CD52 treatment, whereas B cell reduction is transient and can be followed by rebound to higher than pre-treatment frequencies^205-207^.

Anti-CD20

Antibodies that deplete CD20^+^ B cells (e.g., rituximab and the FDA-approved agents ocrelizumab, ublituximab, and ofatumumab) have been widely successful in minimizing MS relapse rate, disability, progression, and lesion loads (volume and frequency). B cell depletion is consistently effective across RRMS and SPMS, is the only recommended treatment for PPMS, and elicits favorable responses in younger patients with inflammatory lesions^15,208-210^. Ocrelizumab treatment notably yields better outcomes than IFNβ treatment across key disease metrics^211^. Recent studies found that ocrelizumab also depletes CD8^+^CD20^+^ T cells and CD8^+^ central memory T cells, including myelin-specific CD8^+^ cells enriched in people with MS^212,213^. CD20-targeted therapy is also noteworthy for the cells it does not deplete – CD138^+^CD20^-^ antibody-secreting cells (ASCs) that develop through B cell differentiation. Accordingly, humoral immune responses are at least partially retained during ocrelizumab treatment.

Treatment Summary and Perspective

The mechanisms of effective immunomodulatory MS treatments are consistent with complex immune interactions at play during neuroinflammatory episodes and progressive CNS injury. B cells are clearly pathogenic effectors in MS, and B cell depletion is the current best practice for disease management. There are many more effective treatment options for RRMS than PPMS or SPMS, which highlights a key priority in developing new therapies^214^. Despite significant improvements, no current DMT can prevent or reverse eventual neurodegeneration^53,215^. As discussed later, current and future therapeutic advances will likely be made by shifting the focus from suppressing pathogenic cells post hoc to preventing MS by addressing its etiologic roots.

**MS Epidemiology and Risk Factors**

Global and Regional Incidence

MS is the most frequent non-trauma related cause of neurologic disability in young adults. An estimated three million people worldwide live with MS in some form, and incidence across geographic regions has risen steadily in recent decades, independent of historical underdiagnosis in some populations^216^. MS is most prevalent in Europe (143 per 100,000 people) and the Americas (118 per 100,000 people, primarily the United States and Canada)^217^, and incidence strongly correlates with latitude (likely due to vitamin D and UV exposure, discussed below). The latitude phenomenon was observed in migration studies of US service members deployed during World War II and the Korean War, wherein travel from higher to lower latitude regions for active duty conferred lower risk for disease^218^. Across a racially and ethnically heterogeneous US population, latitude remains a significant MS risk factor independent of age, race, ethnicity, and sex^219,220^. Although MS prevalence is lower in the Mediterranean (33 per 100,000 people), African countries (9 per 100,000 people), Southeast Asia (9 per 100,000 people), and the Western Pacific (5 per 100,000 people), the confidence of these estimates are in many cases hampered by a lack of data from studies limited to few countries within a given continent or region. However, longitudinal incidence data suggest that MS rates are rising in regions across the globe^217^. Efforts to better understand the complex genetics of MS across global populations will be an especially important focus area for future research^221^.

Age of Onset and Disability

MS onset is most common between the ages of twenty and thirty^222^, and the majority of people living with MS are 55-64 years old^223^. Age at clinical onset is considered to be the primary predictive factor for disease progression and time to reach disability milestones^39^. The relatively narrow age range for onset strongly implicates age-dependent variables including total lifetime exposure to environmental stimuli and changes in cellular function of frequency.

MS Prevalence by Race and Ethnicity

Historically, MS has been viewed as a disease that primarily affects White people. The demographics of countries with high versus low MS prevalence superficially appear consistent with this theory. However, this is partially attributable to significant underdiagnosis of MS in non-White patients stemming from underenrollment in clinical trials and different clinical presentations of symptoms by race. These factors culminate in substantial racial disparities in MS clinical outcomes^224,225^. Recent epidemiologic studies conducted in the US convincingly challenge the conventional view: retrospective analyses of several million electronic health records in Southern California found that MS is equally if not more prevalent in Black individuals than White individuals, while prevalence is lower among Hispanic and Asian people. Black women appear to be at greatest risk for MS^226,227^. Overall, MS cohorts in the US are becoming increasingly ethnically and racially heterogeneous^220,228^.

Racial and ethnic disparities in MS clinical course and disease severity also exist. Black and Hispanic individuals experience more severe effects, as evidenced by younger age at diagnosis and earlier time to disability^229-231^. For example, Black Americans with MS typically present with higher lesion burden at baseline, faster clinical progression, and elevated disease-associated mortality at younger ages^225^. While Black people with MS benefit from DMTs, later diagnosis and social determinants of health contribute to poorer outcomes as measured by greater disability in this population^232^.

Sex Bias in MS Susceptibility

Arguably, the most prominent epidemiologic aspect of MS is the 3:1 female bias in prevalence, which is conserved or even further skewed when stratified by race and ethnicity^220,226,228,233^. Anecdotally, this ratio matches surprisingly well with Charcot’s initial observed cases of ‘la sclérose en plaques’ at Salpêtrière (25 female versus 9 male). Despite elevated risk, females with MS generally reach disability endpoints later in life than males^43^. The female bias in MS is consistent with skews observed in autoimmune disorders including systemic lupus erythematosus (SLE), rheumatoid arthritis (RA), primary Sjögren’s Syndrome (pSS), and myasthenia gravis (MG)^234^. The consistency of female predisposition to autoimmunity clearly implicates sex-specific differences in immune composition or function mediated at the genetic level.

An obvious culprit is X chromosome dosage effects, specifically within the 15% of X-encoded genes that escape X-linked inactivation in females^235^. For example, the single-stranded RNA (ssRNA)-sensing Toll-like receptor encoded by *TLR7* is one such X-linked inactivation escapee that contributes to immune cell activation and autoreactive responses^236,237^. Likewise, *CXCR3* is an X chromosomal gene that encodes a receptor for the inflammatory chemokines; can be expressed in biallelic fashion^238^; and is expressed by neuroinvasive B cells in MS^239^. However, MS genetic susceptibility extends well beyond X chromosomes.

Genetic Predisposition

In 1896, the physician Hermann Eichhorst wrote the first known report of a familial cluster of MS cases, from which he prematurely concluded that the disease was transmitted by heredity^240^. However, studies in twins versus siblings and half-siblings as well as familial case clusters show that elements of MS risk are certainly heritable^241-243^. Though MS is not a simple Mendelian trait, significantly higher disease concordance in monozygotic versus fraternal twins (25-30% versus 2-5%) clearly demonstrates a genetic component^241^. Recent data from mono- and dizygotic twins have delineated contributions of heritable and non-heritable factors in susceptibility^244^.

Many risk loci are proximal to genes involved in immune response induction, modulation, and resolution, and the number of known MS susceptibility genes has grown substantially over the past decade^245-247^.*** The strongest genetic risk comes from the DRB1*1501 allele of the MHC class II gene HLA-DRB1^248^, which accounts for an estimated 17-60% of known genetic susceptibility for MS and strongly implicates molecular interactions in antigen presentation and/or recognition^39,249^. Notably, no single gene accounts for more than 1-2% of MS risk. Thus, MS is a complex genetic disease in the sense that many genes with mild or moderate risk alleles compose a broader susceptibility profile based in interactions with environmental variables^39^.

Environmental Risks

Diet, UV radiation, climate, and exposure to metals and organic solvents are all associated with MS risk. Vitamin D deficiency and smoking have particularly strong disease associations and are noteworthy for their interactions with genetic risk loci and immune system dysfunction^250,251^.

Vitamin D

MS risk is increased in people who have vitamin D deficiency owing to diet and limited sun exposure or impaired vitamin D responses due to mutations in the vitamin D receptor (VDR)^250,252^. The MS latitude gradient is closely related to annual variations in total UV-B exposure and vitamin D levels. It is challenging to fully decouple the protective roles of vitamin D and UV-B exposure due to UV-B dependent D_3_ biosynthesis, however UV-B may have vitamin D-independent effects in reducing MS risk^253^. Intriguingly, MS incidence is significantly lower among people born in November versus May in the northern hemisphere; this relationship is inverted for people born in the southern hemisphere^254,255^. Relative MS risk by hemisphere and birthdate may be related to maternal sun exposure in the first trimester or sun exposure during infancy.

Dietary intake of vitamin D has a significant protective effect in women (relative MS risk = 0.59 versus no supplemental vitamin D)^256^. People with higher circulating levels of 25-OH vitamin D_3_ – especially children and adolescents – likewise have lower MS risk^257^. Beyond reduced incidence, higher serum levels of 25-OH vitamin D_3_ in people diagnosed with CIS are associated with two-fold reduced the risk for conversion to clinically definite MS^258^.

Transcriptional regulation can be substantially altered by vitamin D availability and metabolism. The promoter for the MHC class II risk allele HLA-DRB1*1501 contains a vitamin D response element (VDRE), indicating that vitamin D levels regulate antigen presentation^259^. Genome-wide expression profiling of a longitudinal MS cohort identified a network of VRDE-regulated immunomodulatory genes, many of which overlap with known IFNβ target genes. Expression of genes within this network was associated with higher 25-OH vitamin D_3_ serum levels, reduced disease activity, and fewer Gd-enhancing lesions, whereas MS patients with lower 25-OH vitamin D_3_ levels and higher lesion burdens exhibited elevated expression of genes associated with activated immune responses (TNFSF13B / BAFF, TLR7), immune cell survival (TNFAIP3 / A20, LMO2, CD274 / PD-L1), and inflammatory cytokines (CXCL10, CCL2, CCL8)^260^. Another MS susceptibility gene, CYP24A1, encodes a hydroxylase that converts 25-OH vitamin D_3_ to an inactive metabolite, thereby limiting its bioavailability in cases of enzymatic hyperactivity^261^.

Vitamin D and UV-B can directly regulate immune cell responses. For example, UV exposure induces immune suppressive T regulatory cells (T_reg_) that secrete IL-10^262,263^. IL-10 limits excessive B cell expansion in germinal centers (GCs) by inducing plasma cell differentiation^264^. In mice, UV irradiation induces lymph node (LN)-resident immunoregulatory B cells (B_regs_), which also secrete IL-10 and may promote immune tolerance by suppressing dendritic cell (DC) activation and T cell responses^265-267^. B_reg_-derived IL-10 also limits antiviral responses of CD8^+^ T cells in mice^268^, and IL-10-producing B_reg_ cells are depleted in people with MS during relapses but not remission^269^. Thus, the protective effects of vitamin D and UV-B exposure stem from gene-environment interactions important for suppressing inflammation and resolving immune reactions.

Smoking

People with any history of smoking experience a 1.3-fold hazard for MS onset and a 3.6-fold hazard for progression from RRMS to SPMS after diagnosis versus those who have never smoked^270^. Moreover, people with a history of smoking who ceased upon diagnosis with CIS have reduced risk for progression to clinically definite MS versus those who continued to smoke post diagnosis^271^. People with MS who regularly smoke experience more frequent disease activity (e.g., relapses) even with DMT, higher CNS lesion burden, more extensive BBB disruption and neurodegeneration, and greater disability than non-smokers with MS. Byproducts of smoking (carbon monoxide, free radicals, and cyanates) may also directly induce neurotoxicity^272-275^. Smokers also exhibit elevated C-reactive protein, fibrinogen, and white cell counts^276^. Perivascular leakage of fibrinogen induces microglial activation and ROS generation leading to neuronal damage^277,278^. Excess fibrinogen can also exacerbate neuroinflammatory demyelination by inducing chemokine responses, enhancing antigen presentation, and impeding remyelination by blocking ODP differentiation^279,280^.

MS risk from smoking does not appear to be derived from nicotine itself based on the significant association with smoking but not snuff use (oral smokeless tobacco)^281^. Nicotine may in fact suppress CD4^+^ T cell neuroinflammatory responses by activating nicotinic acetylcholine receptor-mediated repression of TNFα, IFNγ, and T-bet as well as reduced NF-κB transcriptional activity^282^. Evidence for disease-disposing insults within smoke is corroborated by an increased MS risk for non-smokers due to secondhand smoke exposure^283^.

Other Environmental and Lifestyle Factors

Although unrelated to risk for MS onset, heat is an important factor that affects quality of life for people with MS. Excessive heat exposure has long been known to worsen symptoms and affect patient outcomes (Uhthoff’s phenomenon)^284^. Examination of other environmental and lifestyle factors in MS risk – for example, the apparent protection conferred by exposure to cats^285,286^ – is left to the discretion of the curious reader. Certain environmental factors clearly protect against MS risk through gene-environment interactions, while chronic environmental insults can trigger pathologic neuroinflammation. Although the epidemiologic components summarized above must be synthesized to understand MS as an incredibly complex disease with numerous predisposing and protective factors, such consideration does little to answer the foundational question – what causes MS, and how? The main parts of this survey focus on this question, which requires consideration of one last source of risk: infection.

**Footnotes**

* The co-presentation of intention tremor, nystagmus, and dysarthria are indicative, but not dispositive, criteria in the diagnosis of MS, since these symptoms can also be present in other neurologic diseases.

** While not specific to MS, CSF-restricted OCB is one of the most consistent diagnostic features.

*** Notable immune-related genes associated with MS risk loci include: *TNFRSF1A, CD6, IRF8, CD58, SLAMF7, STAT4, CCR4, IKZF1, CXCR5, TRAF3, MAPK3, FCRL1, CD86, IL12A, IL7R, EOMES< IL2RA, TNFAIP3, CD69, CD40, and TNFSF14*.

**References**

1 Eliot, T. S. *Little Gidding*. (Faber & Faber London, 1943).

2 Holmøy, T. A Norse contribution to the history of neurological diseases. *European neurology* **55**, 57-58 (2006).

3 Poser, C. M. The dissemination of multiple sclerosis: a Viking saga? A historical essay. *Annals of Neurology: Official Journal of the American Neurological Association and the Child Neurology Society* **36**, S231-S243 (1994).

4 Murray, T. J. *Multiple sclerosis: the history of a disease*. (Demos medical publishing, 2004).

5 Gough, R. *The Antiquities and Memoirs of the Parish of Myddle, County of Salop*. (Cambridge University Press, 2015).

6 Firth, D. (SAGE Publications, 1941).

7 Carswell, R. *Pathological anatomy: illustrations of the elementary forms of disease*. (Longman, Orme, Brown, Green and Longman, 1838).

8 Gardham, J. *Robert Carswell — Pathological Anatomy: Illustrations of the Elementary Forms of Disease*, <<https://www.gla.ac.uk/myglasgow/library/files/special/exhibns/month/oct2003.html>> (2003).

9 Cruveilhier, J. *Anatomie pathologique du corps humain; ou, Descriptions, avec figures lithographiées et coloriées, des diverses altérations morbides dont le corps humain est susceptible*. (Chez JB Baillière, 1842).

10 Flamm, E. S. THE NEUROLOGY OF JEAN CRUVEILHIER. *Medical History* **17**, 343-355, doi:10.1017/S0025727300018950 (1973).

11 Compston, A. The 150th anniversary of the first depiction of the lesions of multiple sclerosis. *J Neurol Neurosurg Psychiatry* **51**, 1249-1252, doi:10.1136/jnnp.51.10.1249 (1988).

12 Rindfleisch, E. Histologisches Detail zu der grauen Degeneration von Gehirn und Rückenmark.(Zugleich ein Beitrag zu der Lehre von der Entstehung und Verwandlung der Zelle.). *Archiv für pathologische Anatomie und Physiologie und für klinische Medicin* **26**, 474-483 (1863).

13 Clymer, M. *Notes on the Physiology and Pathology of the Nervous System: With Reference to Clinical Medicine. A.--Disseminated Sclerosis of the Brain and Spinal Cord. B.--Annular Or Cortical Sclerois of the Spinal Cord. By Meredith Clymer*. (D. Appleton, 1870).

14 Charcot, J. Histologie de la sclerose en plaques. *Gazette Hôpitaux* **41**, 554 (1868).

15 Hauser, S. L. & Cree, B. A. Treatment of multiple sclerosis: a review. *The American journal of medicine* **133**, 1380-1390. e1382 (2020).

16 Edwards, A. *Disseminated sclerosis*, Victoria University, (1895).

17 Gafson, A., Giovannoni, G. & Hawkes, C. H. The diagnostic criteria for multiple sclerosis: From Charcot to McDonald. *Multiple sclerosis and related disorders* **1**, 9-14 (2012).

18 Marcus, J. F. & Waubant, E. L. Updates on clinically isolated syndrome and diagnostic criteria for multiple sclerosis. *The Neurohospitalist* **3**, 65-80 (2013).

19 Schumacher, G. A. *et al.* Problems of experimental trials of therapy in multiple sclerosis: report by the panel on the evaluation of experimental trials of therapy in multiple sclerosis. *Annals of the New York Academy of Sciences* **122**, 552-568 (1965).

20 EA, K. & DA, F. A study of the crystalline albumin, gamma globulin and total protein in the cerebrospinal fluid of 100 cases of multiple sclerosis and in other diseases. *The American journal of the medical sciences* **219**, 55-64 (1950).

21 Poser, C. M. *et al.* New diagnostic criteria for multiple sclerosis: guidelines for research protocols. *Annals of Neurology: Official Journal of the American Neurological Association and the Child Neurology Society* **13**, 227-231 (1983).

22 Andersson, M. *et al.* Cerebrospinal fluid in the diagnosis of multiple sclerosis: a consensus report. *Journal of Neurology, Neurosurgery & Psychiatry* **57**, 897-902 (1994).

23 McDonald, W. I. *et al.* Recommended diagnostic criteria for multiple sclerosis: guidelines from the International Panel on the diagnosis of multiple sclerosis. *Annals of Neurology: Official Journal of the American Neurological Association and the Child Neurology Society* **50**, 121-127 (2001).

24 Polman, C. H. *et al.* Diagnostic criteria for multiple sclerosis: 2005 revisions to the “McDonald Criteria”. *Annals of Neurology: Official Journal of the American Neurological Association and the Child Neurology Society* **58**, 840-846 (2005).

25 Polman, C. H. *et al.* Diagnostic criteria for multiple sclerosis: 2010 revisions to the McDonald criteria. *Annals of neurology* **69**, 292-302 (2011).

26 Thompson, A. J. *et al.* Diagnosis of multiple sclerosis: 2017 revisions of the McDonald criteria. *The Lancet Neurology* **17**, 162-173 (2018).

27 Ning, L. & Wang, B. Neurofilament light chain in blood as a diagnostic and predictive biomarker for multiple sclerosis: A systematic review and meta-analysis. *Plos one* **17**, e0274565 (2022).

28 Ferreira-Atuesta, C., Reyes, S., Giovanonni, G. & Gnanapavan, S. The evolution of neurofilament light chain in multiple sclerosis. *Frontiers in neuroscience* **15**, 642384 (2021).

29 Bjornevik, K. *et al.* Serum neurofilament light chain levels in patients with presymptomatic multiple sclerosis. *JAMA neurology* **77**, 58-64 (2020).

30 Huang, J. *et al.* Inflammation-related plasma and CSF biomarkers for multiple sclerosis. *Proceedings of the National Academy of Sciences* **117**, 12952-12960 (2020).

31 Housley, W. J., Pitt, D. & Hafler, D. A. Biomarkers in multiple sclerosis. *Clinical immunology* **161**, 51-58 (2015).

32 Marie, P. *Leçons sur les maladies de la moelle*. (G. Masson, 1892).

33 Miller, D., Barkhof, F., Montalban, X., Thompson, A. & Filippi, M. Clinically isolated syndromes suggestive of multiple sclerosis, part I: natural history, pathogenesis, diagnosis, and prognosis. *The Lancet Neurology* **4**, 281-288 (2005).

34 Kurtzke, J. F. Rating neurologic impairment in multiple sclerosis: an expanded disability status scale (EDSS). *Neurology* **33**, 1444-1444 (1983).

35 Barkhof, F. *et al.* Comparison of MRI criteria at first presentation to predict conversion to clinically definite multiple sclerosis. *Brain: a journal of neurology* **120**, 2059-2069 (1997).

36 Tintoré, M. *et al.* Baseline MRI predicts future attacks and disability in clinically isolated syndromes. *Neurology* **67**, 968-972 (2006).

37 Miller, D. H., Chard, D. T. & Ciccarelli, O. Clinically isolated syndromes. *The Lancet Neurology* **11**, 157-169 (2012).

38 Kuhle, J. *et al.* Conversion from clinically isolated syndrome to multiple sclerosis: a large multicentre study. *Multiple Sclerosis Journal* **21**, 1013-1024 (2015).

39 Hauser, S. L. & Oksenberg, J. R. The neurobiology of multiple sclerosis: genes, inflammation, and neurodegeneration. *Neuron* **52**, 61-76 (2006).

40 Brex, P. A. *et al.* A longitudinal study of abnormalities on MRI and disability from multiple sclerosis. *New England Journal of Medicine* **346**, 158-164 (2002).

41 Goodin, D. S. *et al.* Relapses in multiple sclerosis: Relationship to disability. *Multiple sclerosis and related disorders* **6**, 10-20 (2016).

42 Miller, D. H. & Leary, S. M. Primary-progressive multiple sclerosis. *The Lancet Neurology* **6**, 903-912 (2007).

43 Confavreux, C. & Vukusic, S. Natural history of multiple sclerosis: a unifying concept. *Brain* **129**, 606-616 (2006).

44 Kutzelnigg, A. *et al.* Cortical demyelination and diffuse white matter injury in multiple sclerosis. *Brain* **128**, 2705-2712 (2005).

45 Schupfer, P. Ueber die infantile Herdsklerose mit Betrachtungen über sekundäre Degenerationen bei disseminierter Sklerose. Klinische und anatomisch-pathologische Untersuchungen. *Monatsschrift für Psychiatrie und Neurologie* **12**, 60-70 (1902).

46 Boiko, A., Vorobeychik, G., Paty, D., Devonshire, V. & Sadovnick, D. Early onset multiple sclerosis: a longitudinal study. *Neurology* **59**, 1006-1010 (2002).

47 Alroughani, R. & Boyko, A. Pediatric multiple sclerosis: a review. *BMC neurology* **18**, 1-8 (2018).

48 Gorman, M. P., Healy, B. C., Polgar-Turcsanyi, M. & Chitnis, T. Increased relapse rate in pediatric-onset compared with adult-onset multiple sclerosis. *Archives of neurology* **66**, 54-59 (2009).

49 Simon, J. H. & Kleinschmidt-DeMasters, B. K. Variants of multiple sclerosis. *Neuroimaging Clinics of North America* **18**, 703-716 (2008).

50 Baló, J. Encephalitis periaxialis concentrica. *Archives of Neurology & Psychiatry* **19**, 242-264 (1928).

51 Schilder, P. Zur Kenntnis der sogenannten diffusen Sklerose.(Über Encephalitis periaxialis diffusa.). *Zeitschrift für die gesamte Neurologie und Psychiatrie* **10**, 1-60 (1912).

52 Lucchinetti, C. F. *et al.* Clinical and radiographic spectrum of pathologically confirmed tumefactive multiple sclerosis. *Brain* **131**, 1759-1775 (2008).

53 Dendrou, C. A., Fugger, L. & Friese, M. A. Immunopathology of multiple sclerosis. *Nature Reviews Immunology* **15**, 545-558 (2015).

54 Marburg, O. *Die sogenannte akute multiple Sklerose*. (Рипол Классик, 1906).

55 Tenembaum, S., Chitnis, T., Ness, J. & Hahn, J. S. Acute disseminated encephalomyelitis. *Neurology* **68**, S23-S36 (2007).

56 Devic, C. Myelite subaigue compliquee de neurite optique. *Bull Med* **35**, 18-30 (1894).

57 Wingerchuk, D. M., Hogancamp, W. F., O’brien, P. C. & Weinshenker, B. G. The clinical course of neuromyelitis optica (Devic’s syndrome). *Neurology* **53**, 1107-1107 (1999).

58 Bot, J. C. *et al.* Spinal cord abnormalities in recently diagnosed MS patients: added value of spinal MRI examination. *Neurology* **62**, 226-233 (2004).

59 Absinta, M. *et al.* Association of chronic active multiple sclerosis lesions with disability in vivo. *JAMA neurology* **76**, 1474-1483 (2019).

60 Elliott, C. *et al.* Chronic white matter lesion activity predicts clinical progression in primary progressive multiple sclerosis. *Brain* **142**, 2787-2799 (2019).

61 Serafini, B., Rosicarelli, B., Magliozzi, R., Stigliano, E. & Aloisi, F. Detection of ectopic B‐cell follicles with germinal centers in the meninges of patients with secondary progressive multiple sclerosis. *Brain pathology* **14**, 164-174 (2004).

62 Ahmed, S. M. *et al.* Accumulation of meningeal lymphocytes correlates with white matter lesion activity in progressive multiple sclerosis. *JCI insight* **7** (2022).

63 Esiri, M. Immunoglobulin-containing cells in multiple-sclerosis plaques. *The Lancet* **310**, 478-480 (1977).

64 Kidd, D. *et al.* Cortical lesions in multiple sclerosis. *Brain* **122**, 17-26 (1999).

65 Peterson, J. W., Bö, L., Mörk, S., Chang, A. & Trapp, B. D. Transected neurites, apoptotic neurons, and reduced inflammation in cortical multiple sclerosis lesions. *Annals of Neurology: Official Journal of the American Neurological Association and the Child Neurology Society* **50**, 389-400 (2001).

66 Selmaj, K., Raine, C. S., Cannella, B. & Brosnan, C. F. Identification of lymphotoxin and tumor necrosis factor in multiple sclerosis lesions. *The Journal of clinical investigation* **87**, 949-954 (1991).

67 Gay, D. & Esiri, M. Blood-brain barrier damage in acute multiple sclerosis plaques: an immunocytological study. *Brain* **114**, 557-572 (1991).

68 Simpson, J., Newcombe, J., Cuzner, M. & Woodroofe, M. Expression of monocyte chemoattractant protein-1 and other β-chemokines by resident glia and inflammatory cells in multiple sclerosis lesions. *Journal of neuroimmunology* **84**, 238-249 (1998).

69 Schirmer, L. *et al.* Neuronal vulnerability and multilineage diversity in multiple sclerosis. *Nature* **573**, 75-82 (2019).

70 Owens, T. The enigma of multiple sclerosis: inflammation and neurodegeneration cause heterogeneous dysfunction and damage. *Current opinion in neurology* **16**, 259-265 (2003).

71 Wootla, B., Eriguchi, M. & Rodriguez, M. Is multiple sclerosis an autoimmune disease? *Autoimmune diseases* **2012** (2012).

72 Waxman, S. *Multiple sclerosis as a neuronal disease*. (Elsevier, 2005).

73 McFarland, H. F. & Martin, R. Multiple sclerosis: a complicated picture of autoimmunity. *Nature immunology* **8**, 913-919 (2007).

74 Kremenchutzky, M., Rice, G., Baskerville, J., Wingerchuk, D. & Ebers, G. The natural history of multiple sclerosis: a geographically based study 9: observations on the progressive phase of the disease. *Brain* **129**, 584-594 (2006).

75 Traugott, U., Reinherz, E. L. & Raine, C. S. Multiple sclerosis: distribution of T cell subsets within active chronic lesions. *Science* **219**, 308-310 (1983).

76 Zamvil, S. *et al.* T-cell clones specific for myelin basic protein induce chronic relapsing paralysis and demyelination. *Nature* **317**, 355-358 (1985).

77 Zhang, J. *et al.* Increased frequency of interleukin 2-responsive T cells specific for myelin basic protein and proteolipid protein in peripheral blood and cerebrospinal fluid of patients with multiple sclerosis. *The Journal of experimental medicine* **179**, 973-984 (1994).

78 Windhagen, A. *et al.* Expression of costimulatory molecules B7-1 (CD80), B7-2 (CD86), and interleukin 12 cytokine in multiple sclerosis lesions. *The Journal of experimental medicine* **182**, 1985-1996 (1995).

79 Koguchi, K. *et al.* Dysregulated T cell expression of TIM3 in multiple sclerosis. *The Journal of experimental medicine* **203**, 1413-1418 (2006).

80 Olsson, T. *et al.* Autoreactive T lymphocytes in multiple sclerosis determined by antigen-induced secretion of interferon-gamma. *The Journal of clinical investigation* **86**, 981-985 (1990).

81 Sørensen, T. L. *et al.* Expression of specific chemokines and chemokine receptors in the central nervous system of multiple sclerosis patients. *The Journal of clinical investigation* **103**, 807-815 (1999).

82 Pender, M. P. Genetically determined failure of activation-induced apoptosis of autoreactive T cells as a cause of multiple sclerosis. *The Lancet* **351**, 978-981 (1998).

83 Pender, M. P. The pathogenesis of primary progressive multiple sclerosis: antibody-mediated attack and no repair? *Journal of Clinical Neuroscience* **11**, 689-692 (2004).

84 Riedhammer, C. & Weissert, R. Antigen presentation, autoantigens, and immune regulation in multiple sclerosis and other autoimmune diseases. *Frontiers in immunology* **6**, 145196 (2015).

85 Wagner, C. A., Roqué, P. J. & Goverman, J. M. Pathogenic T cell cytokines in multiple sclerosis. *Journal of Experimental Medicine* **217** (2020).

86 Genain, C. P., Cannella, B., Hauser, S. L. & Raine, C. S. Identification of autoantibodies associated with myelin damage in multiple sclerosis. *Nature medicine* **5**, 170-175 (1999).

87 Corcione, A. *et al.* Recapitulation of B cell differentiation in the central nervous system of patients with multiple sclerosis. *Proceedings of the National Academy of Sciences* **101**, 11064-11069 (2004).

88 Bar-Or, A. *et al.* Abnormal B-cell cytokine responses a trigger of T-cell–mediated disease in MS? *Annals of Neurology* **67**, 452-461, doi:<https://doi.org/10.1002/ana.21939> (2010).

89 Michel, L., Touil, H., Pikor, N. B., Gommerman, J. L. & Bar-Or, A. B cells in the multiple sclerosis central nervous system: trafficking and contribution to CNS-compartmentalized inflammation. *Frontiers in immunology* **6**, 171281 (2015).

90 Li, R., Rezk, A., Healy, L. M., Gommerman, J. L. & Bar-Or, A. Cytokine-defined B cell responses as therapeutic targets in multiple sclerosis. *Frontiers in Immunology* **6**, 167192 (2016).

91 Rojas, O. L. *et al.* Recirculating intestinal IgA-producing cells regulate neuroinflammation via IL-10. *Cell* **176**, 610-624. e618 (2019).

92 Trapp, B. D. *et al.* Axonal transection in the lesions of multiple sclerosis. *New England Journal of Medicine* **338**, 278-285 (1998).

93 Trebst, C. *et al.* CCR1+/CCR5+ mononuclear phagocytes accumulate in the central nervous system of patients with multiple sclerosis. *The American journal of pathology* **159**, 1701-1710 (2001).

94 Kouwenhoven, M., Teleshova, N., Özenci, V., Press, R. & Link, H. Monocytes in multiple sclerosis: phenotype and cytokine profile. *Journal of neuroimmunology* **112**, 197-205 (2001).

95 Hendriks, J. J., Teunissen, C. E., de Vries, H. E. & Dijkstra, C. D. Macrophages and neurodegeneration. *Brain Research Reviews* **48**, 185-195 (2005).

96 Boven, L. A. *et al.* Myelin-laden macrophages are anti-inflammatory, consistent with foam cells in multiple sclerosis. *Brain* **129**, 517-526 (2006).

97 He, F. & Sun, Y. E. Glial cells more than support cells? *The international journal of biochemistry & cell biology* **39**, 661-665 (2007).

98 Choi, S. R. *et al.* Meningeal inflammation plays a role in the pathology of primary progressive multiple sclerosis. *Brain* **135**, 2925-2937 (2012).

99 Friese, M. A., Schattling, B. & Fugger, L. Mechanisms of neurodegeneration and axonal dysfunction in multiple sclerosis. *Nature Reviews Neurology* **10**, 225-238 (2014).

100 Gebicke-Haerter, P. J. Microglia in neurodegeneration: Molecular aspects. *Microscopy Research and Technique* **54**, 47-58, doi:<https://doi.org/10.1002/jemt.1120> (2001).

101 Banati, R. B. *et al.* The peripheral benzodiazepine binding site in the brain in multiple sclerosis: Quantitative in vivo imaging of microglia as a measure of disease activity. *Brain* **123**, 2321-2337, doi:10.1093/brain/123.11.2321 (2000).

102 Hickman, S., Izzy, S., Sen, P., Morsett, L. & El Khoury, J. Microglia in neurodegeneration. *Nature neuroscience* **21**, 1359-1369 (2018).

103 Olson, J. K. & Miller, S. D. Microglia initiate central nervous system innate and adaptive immune responses through multiple TLRs. *The Journal of Immunology* **173**, 3916-3924 (2004).

104 Olson, J. K., Girvin, A. M. & Miller, S. D. Direct activation of innate and antigen-presenting functions of microglia following infection with Theiler's virus. *Journal of virology* **75**, 9780-9789 (2001).

105 Kremlev, S. G., Roberts, R. L. & Palmer, C. Differential expression of chemokines and chemokine receptors during microglial activation and inhibition. *Journal of neuroimmunology* **149**, 1-9 (2004).

106 Jack, C., Ruffini, F., Bar‐Or, A. & Antel, J. P. Microglia and multiple sclerosis. *Journal of neuroscience research* **81**, 363-373 (2005).

107 Haider, L. *et al.* Oxidative damage in multiple sclerosis lesions. *Brain* **134**, 1914-1924 (2011).

108 Aloisi, F., Ria, F., Penna, G. & Adorini, L. Microglia are more efficient than astrocytes in antigen processing and in Th1 but not Th2 cell activation. *The Journal of Immunology* **160**, 4671-4680 (1998).

109 Rose, J. W., Hill, K. E., Watt, H. E. & Carlson, N. G. Inflammatory cell expression of cyclooxygenase-2 in the multiple sclerosis lesion. *Journal of neuroimmunology* **149**, 40-49 (2004).

110 Hill, K. E., Zollinger, L. V., Watt, H. E., Carlson, N. G. & Rose, J. W. Inducible nitric oxide synthase in chronic active multiple sclerosis plaques: distribution, cellular expression and association with myelin damage. *Journal of neuroimmunology* **151**, 171-179 (2004).

111 Miron, V. E. *et al.* M2 microglia and macrophages drive oligodendrocyte differentiation during CNS remyelination. *Nature neuroscience* **16**, 1211-1218 (2013).

112 Yamasaki, R. *et al.* Differential roles of microglia and monocytes in the inflamed central nervous system. *Journal of Experimental Medicine* **211**, 1533-1549 (2014).

113 Dong, Y. & Yong, V. W. When encephalitogenic T cells collaborate with microglia in multiple sclerosis. *Nature Reviews Neurology* **15**, 704-717 (2019).

114 Raivich, G. & Banati, R. Brain microglia and blood-derived macrophages: molecular profiles and functional roles in multiple sclerosis and animal models of autoimmune demyelinating disease. *Brain Research Reviews* **46**, 261-281 (2004).

115 Li, Q. & Barres, B. A. Microglia and macrophages in brain homeostasis and disease. *Nature Reviews Immunology* **18**, 225-242 (2018).

116 Mishra, M. K. & Yong, V. W. Myeloid cells—targets of medication in multiple sclerosis. *Nature Reviews Neurology* **12**, 539-551 (2016).

117 Correale, J. & Farez, M. F. The role of astrocytes in multiple sclerosis progression. *Frontiers in neurology* **6**, 153234 (2015).

118 Lennon , V. A., Kryzer , T. J., Pittock , S. J., Verkman , A. S. & Hinson , S. R. IgG marker of optic-spinal multiple sclerosis binds to the aquaporin-4 water channel. *Journal of Experimental Medicine* **202**, 473-477, doi:10.1084/jem.20050304 (2005).

119 Brosnan, C. F. & Raine, C. S. The astrocyte in multiple sclerosis revisited. *Glia* **61**, 453-465 (2013).

120 Argaw, A. T., Gurfein, B. T., Zhang, Y., Zameer, A. & John, G. R. VEGF-mediated disruption of endothelial CLN-5 promotes blood-brain barrier breakdown. *Proceedings of the National Academy of Sciences* **106**, 1977-1982 (2009).

121 Argaw, A. T. *et al.* IL-1β regulates blood-brain barrier permeability via reactivation of the hypoxia-angiogenesis program. *The Journal of Immunology* **177**, 5574-5584 (2006).

122 Tanuma, N., Sakuma, H., Sasaki, A. & Matsumoto, Y. Chemokine expression by astrocytes plays a role in microglia/macrophage activation and subsequent neurodegeneration in secondary progressive multiple sclerosis. *Acta neuropathologica* **112**, 195-204 (2006).

123 Krumbholz, M. *et al.* BAFF is produced by astrocytes and up-regulated in multiple sclerosis lesions and primary central nervous system lymphoma. *The Journal of experimental medicine* **201**, 195-200 (2005).

124 Tschen, S. I. *et al.* CNS viral infection diverts homing of antibody‐secreting cells from lymphoid organs to the CNS. *European journal of immunology* **36**, 603-612 (2006).

125 Farina, C., Aloisi, F. & Meinl, E. Astrocytes are active players in cerebral innate immunity. *Trends in immunology* **28**, 138-145 (2007).

126 Touil, H. *et al.* Human central nervous system astrocytes support survival and activation of B cells: implications for MS pathogenesis. *Journal of Neuroinflammation* **15**, 114, doi:10.1186/s12974-018-1136-2 (2018).

127 Sobel, R. A., Mitchell, M. E. & Fondren, G. Intercellular adhesion molecule-1 (ICAM-1) in cellular immune reactions in the human central nervous system. *The American journal of pathology* **136**, 1309 (1990).

128 Gimenez, M. A. T., Sim, J. E. & Russell, J. H. TNFR1-dependent VCAM-1 expression by astrocytes exposes the CNS to destructive inflammation. *Journal of neuroimmunology* **151**, 116-125 (2004).

129 Fünfschilling, U. *et al.* Glycolytic oligodendrocytes maintain myelin and long-term axonal integrity. *Nature* **485**, 517-521 (2012).

130 Fischer, M. T. *et al.* Disease-specific molecular events in cortical multiple sclerosis lesions. *Brain* **136**, 1799-1815 (2013).

131 Chao, C. C., Hu, S. X., Ehrlich, L. & Peterson, P. K. Interleukin-1 and tumor necrosis factor-α synergistically mediate neurotoxicity: involvement of nitric oxide and of N-methyl-D-aspartate receptors. *Brain, behavior, and immunity* **9**, 355-365 (1995).

132 Lee, S. C., Dickson, D. W., Liu, W. & Brosnan, C. F. Induction of nitric oxide synthase activity in human astrocytes by interleukin-1β and interferon-γ. *Journal of neuroimmunology* **46**, 19-24 (1993).

133 Lee, S., Liu, W., Dickson, D., Brosnan, C. & Berman, J. Cytokine production by human fetal microglia and astrocytes. Differential induction by lipopolysaccharide and IL-1 beta. *Journal of immunology (Baltimore, Md.: 1950)* **150**, 2659-2667 (1993).

134 Chung, I. Y. & Benveniste, E. N. Tumor necrosis factor-alpha production by astrocytes. Induction by lipopolysaccharide, IFN-gamma, and IL-1 beta. *Journal of immunology (Baltimore, Md.: 1950)* **144**, 2999-3007 (1990).

135 Liddelow, S. A. *et al.* Neurotoxic reactive astrocytes are induced by activated microglia. *Nature* **541**, 481-487 (2017).

136 Absinta, M. *et al.* A lymphocyte–microglia–astrocyte axis in chronic active multiple sclerosis. *Nature* **597**, 709-714 (2021).

137 Touil, H. *et al.* Cross-talk between B cells, microglia and macrophages, and implications to central nervous system compartmentalized inflammation and progressive multiple sclerosis. *EBioMedicine* **96** (2023).

138 Kasper, L. H. & Reder, A. T. Immunomodulatory activity of interferon‐beta. *Annals of clinical and translational neurology* **1**, 622-631 (2014).

139 Panitch, H., Haley, A., Hirsch, R. & Johnson, K. Exacerbations of multiple sclerosis in patients treated with gamma interferon. *The Lancet* **329**, 893-895 (1987).

140 Waksman, B. H. & Reynolds, W. E. Multiple sclerosis as a disease of immune regulation. *Proceedings of the Society for Experimental Biology and Medicine* **175**, 282-294 (1984).

141 Basham, T. & Merigan, T. C. Recombinant interferon-gamma increases HLA-DR synthesis and expression. *Journal of immunology (Baltimore, Md.: 1950)* **130**, 1492-1494 (1983).

142 Neighbour, P. A., Miller, A. E. & Bloom, B. R. Interferon responses of leukocytes in multiple sclerosis. *Neurology* **31**, 561-561 (1981).

143 Vervliet, G. *et al.* Interferon production and natural killer (NK) activity in leukocyte cultures from multiple sclerosis patients. *Journal of the neurological sciences* **60**, 137-150 (1983).

144 Beck, J. *et al.* Increased production of interferon gamma and tumor necrosis factor precedes clinical manifestation in multiple sclerosis: do cytokines trigger off exacerbations? *Acta Neurologica Scandinavica* **78**, 318-323 (1988).

145 Correale, J. *et al.* Patterns of cytokine secretion by autoreactive proteolipid protein-specific T cell clones during the course of multiple sclerosis. *Journal of immunology (Baltimore, Md.: 1950)* **154**, 2959-2968 (1995).

146 Horwitz, M. S., Evans, C. F., Mcgavern, D. B., Rodriguez, M. & Oldstone, M. B. Primary demyelination in transgenic mice expressing interferon-γ. *Nature medicine* **3**, 1037-1041 (1997).

147 Group, I. M. S. S. Interferon beta‐1b is effective in relapsing‐remitting multiple sclerosis: I. Clinical results of a multicenter, randomized, double‐blind, placebo-controlled trial. *Neurology* **43**, 655-655 (1993).

148 Paty, D., Li, D. B., Group, U. M. M. S. & Group, I. M. S. S. Interferon beta‐1b is effective in relapsing‐remitting multiple sclerosis: II. MRI analysis results of a multicenter, randomized, double‐blind, placebo‐controlled trial. *neurology* **43**, 662-662 (1993).

149 Jacobs, L. D. *et al.* Intramuscular interferon beta‐1a for disease progression in relapsing multiple sclerosis. *Annals of Neurology: Official Journal of the American Neurological Association and the Child Neurology Society* **39**, 285-294 (1996).

150 Yong, V. W., Chabot, S., Stuve, O. & Williams, G. Interferon beta in the treatment of multiple sclerosis: mechanisms of action. *Neurology* **51**, 682-689 (1998).

151 Dhib-Jalbut, S. & Marks, S. Interferon-β mechanisms of action in multiple sclerosis. *Neurology* **74**, S17-S24 (2010).

152 Stone, L. A. *et al.* The effect of interferon‐β on blood—brain barrier disruptions demonstrated by constrast‐enhanced magnetic resonance imaging in relapsing—remitting multiple sclerosis. *Annals of Neurology: Official Journal of the American Neurological Association and the Child Neurology Society* **37**, 611-619 (1995).

153 Noronha, A., Toscas, A. & Jensen, M. A. Interferon β decreases T cell activation and interferon γ production in multiple sclerosis. *Journal of neuroimmunology* **46**, 145-153 (1993).

154 Genc, K., Dona, D. L. & Reder, A. T. Increased CD80 (+) B cells in active multiple sclerosis and reversal by interferon beta-1b therapy. *The Journal of clinical investigation* **99**, 2664-2671 (1997).

155 Jiang, H. *et al.* Interferon β-lb reduces Interferon γ-induced antigen-presenting capacity of human glial and B cells. *Journal of Neuroimmunology* **61**, 17-25, doi:<https://doi.org/10.1016/0165-5728(95)00072-A> (1995).

156 Good-Jacobson, K. L., Song, E., Anderson, S., Sharpe, A. H. & Shlomchik, M. J. CD80 expression on B cells regulates murine T follicular helper development, germinal center B cell survival, and plasma cell generation. *J Immunol* **188**, 4217-4225, doi:10.4049/jimmunol.1102885 (2012).

157 Brinkmann, V. *et al.* The immune modulator FTY720 targets sphingosine 1-phosphate receptors. *Journal of Biological Chemistry* **277**, 21453-21457 (2002).

158 Zemann, B. *et al.* Sphingosine kinase type 2 is essential for lymphopenia induced by the immunomodulatory drug FTY720. *Blood* **107**, 1454-1458 (2006).

159 Albert, R. *et al.* Novel immunomodulator FTY720 is phosphorylated in rats and humans to form a single stereoisomer. Identification, chemical proof, and biological characterization of the biologically active species and its enantiomer. *Journal of medicinal chemistry* **48**, 5373-5377 (2005).

160 Matloubian, M. *et al.* Lymphocyte egress from thymus and peripheral lymphoid organs is dependent on S1P receptor 1. *Nature* **427**, 355-360 (2004).

161 Mandala, S. *et al.* Alteration of lymphocyte trafficking by sphingosine-1-phosphate receptor agonists. *Science* **296**, 346-349 (2002).

162 Alvarez, S. E., Milstien, S. & Spiegel, S. Autocrine and paracrine roles of sphingosine-1-phosphate. *Trends in Endocrinology & Metabolism* **18**, 300-307 (2007).

163 Miron, V. E. *et al.* Fingolimod (FTY720) enhances remyelination following demyelination of organotypic cerebellar slices. *The American journal of pathology* **176**, 2682-2694 (2010).

164 Choi, J. W. *et al.* FTY720 (fingolimod) efficacy in an animal model of multiple sclerosis requires astrocyte sphingosine 1-phosphate receptor 1 (S1P1) modulation. *Proceedings of the National Academy of Sciences* **108**, 751-756 (2011).

165 Jackson, S. J., Giovannoni, G. & Baker, D. Fingolimod modulates microglial activation to augment markers of remyelination. *Journal of neuroinflammation* **8**, 1-12 (2011).

166 Kappos, L. *et al.* A placebo-controlled trial of oral fingolimod in relapsing multiple sclerosis. *New England Journal of Medicine* **362**, 387-401 (2010).

167 Cohen, J. A. *et al.* Oral Fingolimod or Intramuscular Interferon for Relapsing Multiple Sclerosis. *New England Journal of Medicine* **362**, 402-415, doi:10.1056/NEJMoa0907839 (2010).

168 Lublin, F. *et al.* Oral fingolimod in primary progressive multiple sclerosis (INFORMS): a phase 3, randomised, double-blind, placebo-controlled trial. *The Lancet* **387**, 1075-1084 (2016).

169 Bomprezzi, R. Dimethyl fumarate in the treatment of relapsing–remitting multiple sclerosis: an overview. *Therapeutic advances in neurological disorders* **8**, 20-30 (2015).

170 de Jong, R. *et al.* Selective stimulation of T helper 2 cytokine responses by the anti‐psoriasis agent monomethylfumarate. *European journal of immunology* **26**, 2067-2074 (1996).

171 Chen, H. *et al.* Hydroxycarboxylic acid receptor 2 mediates dimethyl fumarate’s protective effect in EAE. *The Journal of clinical investigation* **124**, 2188-2192 (2014).

172 Schimrigk, S. *et al.* Oral fumaric acid esters for the treatment of active multiple sclerosis: an open‐label, baseline‐controlled pilot study. *European journal of neurology* **13**, 604-610 (2006).

173 Kappos, L. *et al.* Efficacy and safety of oral fumarate in patients with relapsing-remitting multiple sclerosis: a multicentre, randomised, double-blind, placebo-controlled phase IIb study. *The Lancet* **372**, 1463-1472 (2008).

174 Gold, R. *et al.* Placebo-controlled phase 3 study of oral BG-12 for relapsing multiple sclerosis. *New England Journal of Medicine* **367**, 1098-1107 (2012).

175 Fox, R. J. *et al.* Placebo-controlled phase 3 study of oral BG-12 or glatiramer in multiple sclerosis. *New England Journal of Medicine* **367**, 1087-1097 (2012).

176 Spencer, C. M., Crabtree-Hartman, E. C., Lehmann-Horn, K., Cree, B. A. & Zamvil, S. S. Reduction of CD8+ T lymphocytes in multiple sclerosis patients treated with dimethyl fumarate. *Neurology-Neuroimmunology Neuroinflammation* **2** (2015).

177 Skulina, C. *et al.* Multiple sclerosis: Brain-infiltrating CD8<sup>+</sup> T cells persist as clonal expansions in the cerebrospinal fluid and blood. *Proceedings of the National Academy of Sciences* **101**, 2428-2433, doi:doi:10.1073/pnas.0308689100 (2004).

178 Friese, M. A. & Fugger, L. Pathogenic CD8+ T cells in multiple sclerosis. *Annals of Neurology* **66**, 132-141, doi:<https://doi.org/10.1002/ana.21744> (2009).

179 Teitelbaum, D., Meshorer, A., Hirshfeld, T., Arnon, R. & Sela, M. Suppression of experimental allergic encephalomyelitis by a synthetic polypeptide. *European journal of immunology* **1**, 242-248 (1971).

180 Racke, M. K., Martin, R., McFarland, H. & Fritz, R. B. Copolymer-1-induced inhibition of antigen-specific T cell activation: interference with antigen presentation. *Journal of neuroimmunology* **37**, 75-84 (1992).

181 Fridkis-Hareli, M. *et al.* Direct binding of myelin basic protein and synthetic copolymer 1 to class II major histocompatibility complex molecules on living antigen-presenting cells--specificity and promiscuity. *Proceedings of the National Academy of Sciences* **91**, 4872-4876 (1994).

182 Chabot, S. *et al.* Cytokine production in T lymphocyte-microglia interaction is attenuated by glatiramer acetate: a mechanism for therapeutic efficacy in multiple sclerosis. *Multiple Sclerosis Journal* **8**, 299-306 (2002).

183 Miller, A. *et al.* Treatment of multiple sclerosis with copolymer-1 (Copaxone®): implicating mechanisms of Th1 to Th2/Th3 immune-deviation. *Journal of neuroimmunology* **92**, 113-121 (1998).

184 Duda, P. W., Schmied, M. C., Cook, S. L., Krieger, J. I. & Hafler, D. A. Glatiramer acetate (Copaxone®) induces degenerate, Th2-polarized immune responses in patients with multiple sclerosis. *The Journal of clinical investigation* **105**, 967-976 (2000).

185 Karandikar, N. J. *et al.* Glatiramer acetate (Copaxone) therapy induces CD8+ T cell responses in patients with multiple sclerosis. *The Journal of clinical investigation* **109**, 641-649 (2002).

186 Tennakoon, D. K. *et al.* Therapeutic induction of regulatory, cytotoxic CD8+ T cells in multiple sclerosis. *The Journal of Immunology* **176**, 7119-7129 (2006).

187 Johnson, K. *et al.* Copolymer 1 reduces relapse rate and improves disability in relapsing‐remitting multiple sclerosis: results of a phase III multicenter, double‐blind, placebo‐controlled trial. *Neurology* **45**, 1268-1276 (1995).

188 Wolinsky, J. S. *et al.* Glatiramer acetate in primary progressive multiple sclerosis: results of a multinational, multicenter, double‐blind, placebo‐controlled trial. *Annals of neurology* **61**, 14-24 (2007).

189 Fox, E. J. Mechanism of action of mitoxantrone. *Neurology* **63**, S15-S18 (2004).

190 Duddy, M. *et al.* Distinct effector cytokine profiles of memory and naive human B cell subsets and implication in multiple sclerosis. *The Journal of Immunology* **178**, 6092-6099 (2007).

191 Fidler, J., DeJoy, S. Q. & Gibbons Jr, J. Selective immunomodulation by the antineoplastic agent mitoxantrone. I. Suppression of B lymphocyte function. *Journal of immunology (Baltimore, Md.: 1950)* **137**, 727-732 (1986).

192 Bellosillo, B., Colomer, D., Pons, G. & Gil, J. Mitoxantrone, a topoisomerase II inhibitor, induces apoptosis of B‐chronic lymphocytic leukaemia cells. *British journal of haematology* **100**, 142-146 (1998).

193 Hartung, H.-P. *et al.* Mitoxantrone in progressive multiple sclerosis: a placebo-controlled, double-blind, randomised, multicentre trial. *The Lancet* **360**, 2018-2025 (2002).

194 Baker, D. *et al.* Both cladribine and alemtuzumab may effect MS via B-cell depletion. *Neurology-Neuroimmunology Neuroinflammation* **4** (2017).

195 Giovannoni, G. *et al.* A placebo-controlled trial of oral cladribine for relapsing multiple sclerosis. *New England Journal of Medicine* **362**, 416-426 (2010).

196 Baker, D., Marta, M., Pryce, G., Giovannoni, G. & Schmierer, K. Memory B cells are major targets for effective immunotherapy in relapsing multiple sclerosis. *EBioMedicine* **16**, 41-50 (2017).

197 Van Oosten, B. *et al.* Treatment of multiple sclerosis with the monoclonal anti-CD4 antibody cM-T412: Results of a randomized, double-blind, placebo-controlled MR-monitored phase II trial. *Neurology* **49**, 351-357 (1997).

198 Rep, M. *et al.* Treatment with depleting CD4 monoclonal antibody results in a preferential loss of circulating naive T cells but does not affect IFN-gamma secreting TH1 cells in humans. *The Journal of clinical investigation* **99**, 2225-2231 (1997).

199 Polman, C. H. *et al.* A randomized, placebo-controlled trial of natalizumab for relapsing multiple sclerosis. *New England Journal of Medicine* **354**, 899-910 (2006).

200 Verbeek, M. M., Westphal, J. R., Ruiter, D. J. & De Waal, R. T lymphocyte adhesion to human brain pericytes is mediated via very late antigen-4/vascular cell adhesion molecule-1 interactions. *Journal of immunology (Baltimore, Md.: 1950)* **154**, 5876-5884 (1995).

201 Greenwood, J., Wang, Y. & Calder, V. Lymphocyte adhesion and transendothelial migration in the central nervous system: the role of LFA-1, ICAM-1, VLA-4 and VCAM-1. off. *Immunology* **86**, 408 (1995).

202 Chabot, S., Williams, G. & Yong, V. W. Microglial production of TNF-alpha is induced by activated T lymphocytes. Involvement of VLA-4 and inhibition by interferonbeta-1b. *The Journal of clinical investigation* **100**, 604-612 (1997).

203 Investigators, C. T. Alemtuzumab vs. interferon beta-1a in early multiple sclerosis. *New England Journal of Medicine* **359**, 1786-1801 (2008).

204 Cohen, J. A. *et al.* Alemtuzumab versus interferon beta 1a as first-line treatment for patients with relapsing-remitting multiple sclerosis: a randomised controlled phase 3 trial. *The Lancet* **380**, 1819-1828 (2012).

205 Barton, J. *et al.* Tumefactive demyelination following treatment for relapsing multiple sclerosis with alemtuzumab. *Neurology* **88**, 1004-1006 (2017).

206 Haghikia, A. *et al.* Severe B-cell-mediated CNS disease secondary to alemtuzumab therapy. *The Lancet Neurology* **16**, 104-106 (2017).

207 Rinaldi, F. *et al.* Evidence of B-cell dysregulation in severe CNS inflammation after alemtuzumab therapy. *Neurology-Neuroimmunology Neuroinflammation* **5** (2018).

208 Hauser, S. L. *et al.* B-cell depletion with rituximab in relapsing–remitting multiple sclerosis. *New England Journal of Medicine* **358**, 676-688 (2008).

209 Kappos, L. *et al.* Ocrelizumab in relapsing-remitting multiple sclerosis: a phase 2, randomised, placebo-controlled, multicentre trial. *The Lancet* **378**, 1779-1787 (2011).

210 Hawker, K. *et al.* Rituximab in patients with primary progressive multiple sclerosis: results of a randomized double‐blind placebo‐controlled multicenter trial. *Annals of neurology* **66**, 460-471 (2009).

211 Hauser, S. L. *et al.* Ocrelizumab versus Interferon Beta-1a in Relapsing Multiple Sclerosis. *New England Journal of Medicine* **376**, 221-234, doi:10.1056/NEJMoa1601277 (2017).

212 Sabatino Jr, J. J. *et al.* Anti-CD20 therapy depletes activated myelin-specific CD8+ T cells in multiple sclerosis. *Proceedings of the National Academy of Sciences* **116**, 25800-25807 (2019).

213 Mathias, A. *et al.* Ocrelizumab Impairs the Phenotype and Function of Memory CD8+ T Cells: A 1-Year Longitudinal Study in Patients With Multiple Sclerosis. *Neurology-Neuroimmunology Neuroinflammation* **10** (2023).

214 Feinstein, A., Freeman, J. & Lo, A. C. Treatment of progressive multiple sclerosis: what works, what does not, and what is needed. *The Lancet Neurology* **14**, 194-207 (2015).

215 Smith, C. & Khanna, R. Adoptive T-cell therapy targeting Epstein–Barr virus as a treatment for multiple sclerosis. *Clinical & Translational Immunology* **12**, e1444, doi:<https://doi.org/10.1002/cti2.1444> (2023).

216 Wallin, M. T. *et al.* Global, regional, and national burden of multiple sclerosis 1990–2016: a systematic analysis for the Global Burden of Disease Study 2016. *The Lancet Neurology* **18**, 269-285 (2019).

217 Walton, C. *et al.* Rising prevalence of multiple sclerosis worldwide: Insights from the Atlas of MS. *Multiple Sclerosis Journal* **26**, 1816-1821 (2020).

218 Kurtzke, J. F., Beebe, G. W. & Norman, J. E. Epidemiology of multiple sclerosis in US veterans: III. Migration and the risk of MIS. *Neurology* **35**, 672-672 (1985).

219 Simpson, S., Blizzard, L., Otahal, P., Van der Mei, I. & Taylor, B. Latitude is significantly associated with the prevalence of multiple sclerosis: a meta-analysis. *Journal of Neurology, Neurosurgery & Psychiatry* **82**, 1132-1141 (2011).

220 Hittle, M. *et al.* Population-Based Estimates for the Prevalence of Multiple Sclerosis in the United States by Race, Ethnicity, Age, Sex, and Geographic Region. *JAMA neurology* (2023).

221 Jacobs, B. M. *et al.* Towards a global view of multiple sclerosis genetics. *Nature Reviews Neurology* **18**, 613-623 (2022).

222 McGinley, M. P., Goldschmidt, C. H. & Rae-Grant, A. D. Diagnosis and Treatment of Multiple Sclerosis: A Review. *JAMA* **325**, 765-779, doi:10.1001/jama.2020.26858 (2021).

223 Wallin, M. T. *et al.* The prevalence of MS in the United States: a population-based estimate using health claims data. *Neurology* **92**, e1029-e1040 (2019).

224 Khan, O. *et al.* Multiple sclerosis in US minority populations: clinical practice insights. *Neurology: Clinical Practice* **5**, 132-142 (2015).

225 Okai, A. F. *et al.* Advancing Care and Outcomes for African American Patients With Multiple Sclerosis. *Neurology* **98**, 1015-1020, doi:10.1212/wnl.0000000000200791 (2022).

226 Langer-Gould, A. M., Gonzales, E. G., Smith, J. B., Li, B. H. & Nelson, L. M. Racial and ethnic disparities in multiple sclerosis prevalence. *Neurology* **98**, e1818-e1827 (2022).

227 Langer-Gould, A., Brara, S. M., Beaber, B. E. & Zhang, J. L. Incidence of multiple sclerosis in multiple racial and ethnic groups. *Neurology* **80**, 1734-1739 (2013).

228 Wallin, M. T. *et al.* The Gulf War era multiple sclerosis cohort: age and incidence rates by race, sex and service. *Brain* **135**, 1778-1785, doi:10.1093/brain/aws099 (2012).

229 Hadjixenofontos, A. *et al.* Clinical expression of multiple sclerosis in Hispanic whites of primarily Caribbean ancestry. *Neuroepidemiology* **44**, 262-268 (2015).

230 Ventura, R. E., Antezana, A. O., Bacon, T. & Kister, I. Hispanic Americans and African Americans with multiple sclerosis have more severe disease course than Caucasian Americans. *Multiple Sclerosis Journal* **23**, 1554-1557 (2017).

231 Amezcua, L., Rivera, V. M., Vazquez, T. C., Baezconde-Garbanati, L. & Langer-Gould, A. Health Disparities, Inequities, and Social Determinants of Health in Multiple Sclerosis and Related Disorders in the US: A Review. *JAMA Neurology* **78**, 1515-1524, doi:10.1001/jamaneurol.2021.3416 (2021).

232 Cipriani, V. P. & Klein, S. Clinical characteristics of multiple sclerosis in African-Americans. *Current Neurology and Neuroscience Reports* **19**, 1-6 (2019).

233 Orton, S.-M. *et al.* Sex ratio of multiple sclerosis in Canada: a longitudinal study. *The Lancet Neurology* **5**, 932-936 (2006).

234 Billi, A. C., Kahlenberg, J. M. & Gudjonsson, J. E. Sex bias in autoimmunity. *Curr Opin Rheumatol* **31**, 53-61, doi:10.1097/bor.0000000000000564 (2019).

235 Carrel, L. & Willard, H. F. X-inactivation profile reveals extensive variability in X-linked gene expression in females. *Nature* **434**, 400-404 (2005).

236 Souyris, M. *et al.* TLR7 escapes X chromosome inactivation in immune cells. *Science immunology* **3**, eaap8855 (2018).

237 Yu, B. *et al.* B cell-specific XIST complex enforces X-inactivation and restrains atypical B cells. *Cell* **184**, 1790-1803. e1717 (2021).

238 Wang, J. *et al.* Unusual maintenance of X chromosome inactivation predisposes female lymphocytes for increased expression from the inactive X. *Proceedings of the National Academy of Sciences* **113**, E2029-E2038 (2016).

239 van Langelaar, J. *et al.* Induction of brain‐infiltrating T‐bet–expressing B cells in multiple sclerosis. *Annals of neurology* **86**, 264-278 (2019).

240 Eichhorst, H. Über infantile und hereditäre multiple Sklerose. *Archiv für pathologische Anatomie und Physiologie und für klinische Medicin* **146**, 173-192 (1896).

241 Ebers, G. C. *et al.* A population-based study of multiple sclerosis in twins. *New England Journal of Medicine* **315**, 1638-1642 (1986).

242 Sadovnick, A., Dyment, D., Ebers, G., Risch, N. & Group, C. C. S. Evidence for genetic basis of multiple sclerosis. *The Lancet* **347**, 1728-1730 (1996).

243 Ebers, G. C. *et al.* The natural history of multiple sclerosis: a geographically based study: 8: Familial multiple sclerosis. *Brain* **123**, 641-649, doi:10.1093/brain/123.3.641 (2000).

244 Ingelfinger, F. *et al.* Twin study reveals non-heritable immune perturbations in multiple sclerosis. *Nature* **603**, 152-158, doi:10.1038/s41586-022-04419-4 (2022).

245 De Jager, P. L. *et al.* Meta-analysis of genome scans and replication identify CD6, IRF8 and TNFRSF1A as new multiple sclerosis susceptibility loci. *Nature Genetics* **41**, 776-782, doi:10.1038/ng.401 (2009).

246 Consortium*†, I. M. S. G. *et al.* Multiple sclerosis genomic map implicates peripheral immune cells and microglia in susceptibility. *Science* **365**, eaav7188, doi:doi:10.1126/science.aav7188 (2019).

247 Consortium, I. I. G. *et al.* Analysis of immune-related loci identifies 48 new susceptibility variants for multiple sclerosis. *Nature genetics* **45**, 1353-1360 (2013).

248 Dyment, D. A., Ebers, G. C. & Sadovnick, A. D. Genetics of multiple sclerosis. *The Lancet Neurology* **3**, 104-110 (2004).

249 Group, M. S. G. *et al.* Linkage of the MHC to familial multiple sclerosis suggests genetic heterogeneity. *Human molecular genetics* **7**, 1229-1234 (1998).

250 Ascherio, A. & Munger, K. L. Environmental risk factors for multiple sclerosis. Part II: Noninfectious factors. *Annals of Neurology* **61**, 504-513, doi:<https://doi.org/10.1002/ana.21141> (2007).

251 Olsson, T., Barcellos, L. F. & Alfredsson, L. Interactions between genetic, lifestyle and environmental risk factors for multiple sclerosis. *Nature Reviews Neurology* **13**, 25-36 (2017).

252 Ascherio, A., Munger, K. L. & Simon, K. C. Vitamin D and multiple sclerosis. *The Lancet Neurology* **9**, 599-612 (2010).

253 Lucas, R. M., Byrne, S. N., Correale, J., Ilschner, S. & Hart, P. H. Ultraviolet radiation, vitamin D and multiple sclerosis. *Neurodegenerative Disease Management* **5**, 413-424, doi:10.2217/nmt.15.33 (2015).

254 Willer, C. J. *et al.* Timing of birth and risk of multiple sclerosis: population based study. *Bmj* **330**, 120 (2005).

255 Staples, J., Ponsonby, A.-L. & Lim, L. Low maternal exposure to ultraviolet radiation in pregnancy, month of birth, and risk of multiple sclerosis in offspring: longitudinal analysis. *Bmj* **340** (2010).

256 Munger, K. L. *et al.* Vitamin D intake and incidence of multiple sclerosis. *Neurology* **62**, 60-65 (2004).

257 Munger, K. L., Levin, L. I., Hollis, B. W., Howard, N. S. & Ascherio, A. Serum 25-hydroxyvitamin D levels and risk of multiple sclerosis. *Jama* **296**, 2832-2838 (2006).

258 Martinelli, V. *et al.* Vitamin D levels and risk of multiple sclerosis in patients with clinically isolated syndromes. *Multiple Sclerosis Journal* **20**, 147-155 (2014).

259 Ramagopalan, S. V. *et al.* Expression of the multiple sclerosis-associated MHC class II Allele HLA-DRB1* 1501 is regulated by vitamin D. *PLoS genetics* **5**, e1000369 (2009).

260 Munger, K. L. *et al.* Molecular mechanism underlying the impact of vitamin D on disease activity of MS. *Annals of clinical and translational neurology* **1**, 605-617 (2014).

261 Control, I. M. S. G. C. W. T. C. Genetic risk and a primary role for cell-mediated immune mechanisms in multiple sclerosis. *Nature* **476**, 214-219 (2011).

262 Schwarz, T. 25 years of UV‐induced immunosuppression mediated by T cells—from disregarded T suppressor cells to highly respected regulatory T cells. *Photochemistry and photobiology* **84**, 10-18 (2008).

263 Chaudhry, A. *et al.* Interleukin-10 signaling in regulatory T cells is required for suppression of Th17 cell-mediated inflammation. *Immunity* **34**, 566-578, doi:10.1016/j.immuni.2011.03.018 (2011).

264 Choe, J. & Choi, Y. S. IL‐10 interrupts memory B cell expansion in the germinal center by inducing differentiation into plasma cells. *European journal of immunology* **28**, 508-515 (1998).

265 Byrne, S. N. & Halliday, G. M. B cells activated in lymph nodes in response to ultraviolet irradiation or by interleukin-10 inhibit dendritic cell induction of immunity. *Journal of Investigative Dermatology* **124**, 570-578 (2005).

266 Matsumura, Y., Byrne, S. N., Nghiem, D. X., Miyahara, Y. & Ullrich, S. E. A role for inflammatory mediators in the induction of immunoregulatory B cells. *The Journal of Immunology* **177**, 4810-4817 (2006).

267 Mauri, C. & Bosma, A. Immune regulatory function of B cells. *Annual review of immunology* **30**, 221-241 (2012).

268 Madan, R. *et al.* Nonredundant roles for B cell-derived IL-10 in immune counter-regulation. *The Journal of Immunology* **183**, 2312-2320 (2009).

269 Knippenberg, S. *et al.* Reduction in IL-10 producing B cells (Breg) in multiple sclerosis is accompanied by a reduced naive/memory Breg ratio during a relapse but not in remission. *Journal of neuroimmunology* **239**, 80-86 (2011).

270 Hernán, M. A. *et al.* Cigarette smoking and the progression of multiple sclerosis. *Brain* **128**, 1461-1465 (2005).

271 van der Vuurst de Vries, R. M. *et al.* Smoking at time of CIS increases the risk of clinically definite multiple sclerosis. *Journal of neurology* **265**, 1010-1015 (2018).

272 Zivadinov, R. *et al.* Smoking is associated with increased lesion volumes and brain atrophy in multiple sclerosis. *Neurology* **73**, 504-510 (2009).

273 Petersen, E. R. *et al.* Smoking is associated with increased disease activity during natalizumab treatment in multiple sclerosis. *Multiple Sclerosis Journal* **25**, 1298-1305 (2019).

274 Petersen, E. R. *et al.* Smoking affects the interferon beta treatment response in multiple sclerosis. *Neurology* **90**, e593-e600 (2018).

275 Rosso, M. & Chitnis, T. Association between cigarette smoking and multiple sclerosis: a review. *JAMA neurology* **77**, 245-253 (2020).

276 Wannamethee, S. G. *et al.* Associations between cigarette smoking, pipe/cigar smoking, and smoking cessation, and haemostatic and inflammatory markers for cardiovascular disease. *European heart journal* **26**, 1765-1773 (2005).

277 Davalos, D. *et al.* Fibrinogen-induced perivascular microglial clustering is required for the development of axonal damage in neuroinflammation. *Nature communications* **3**, 1227 (2012).

278 Petersen, M. A., Ryu, J. K. & Akassoglou, K. Fibrinogen in neurological diseases: mechanisms, imaging and therapeutics. *Nature Reviews Neuroscience* **19**, 283-301 (2018).

279 Ryu, J. K. *et al.* Blood coagulation protein fibrinogen promotes autoimmunity and demyelination via chemokine release and antigen presentation. *Nature communications* **6**, 8164 (2015).

280 Petersen, M. A. *et al.* Fibrinogen activates BMP signaling in oligodendrocyte progenitor cells and inhibits remyelination after vascular damage. *Neuron* **96**, 1003-1012. e1007 (2017).

281 Hedström, A. K., Bäärnhielm, M., Olsson, T. & Alfredsson, L. Tobacco smoking, but not Swedish snuff use, increases the risk of multiple sclerosis. *Neurology* **73**, 696-701 (2009).

282 Nizri, E. *et al.* Activation of the cholinergic anti-inflammatory system by nicotine attenuates neuroinflammation via suppression of Th1 and Th17 responses. *The Journal of Immunology* **183**, 6681-6688 (2009).

283 Hedström, A., Bäärnhielm, M., Olsson, T. & Alfredsson, L. Exposure to environmental tobacco smoke is associated with increased risk for multiple sclerosis. *Multiple Sclerosis Journal* **17**, 788-793 (2011).

284 Kjellstrom, T., Butler, A. J., Lucas, R. M. & Bonita, R. Public health impact of global heating due to climate change: potential effects on chronic non-communicable diseases. *International journal of public health* **55**, 97-103 (2010).

285 Ghadirian, P., Dadgostar, B., Azani, R. & Maisonneuve, P. A case-control study of the association between socio-demographic, lifestyle and medical history factors and multiple sclerosis. *Canadian journal of public health* **92**, 281-285 (2001).

286 Gustavsen, M. W. *et al.* Environmental exposures and the risk of multiple sclerosis investigated in a Norwegian case-control study. *BMC neurology* **14**, 1-8 (2014).
