# Supplementary material for: Extracellular vesicles in malaria: proteomics insights, in vitro and in vivo studies indicate the need for transitioning to natural human infections
Source: mBio. 2025 Jan 27;16(3):e02304-24. doi: 10.1128/mbio.02304-24 (PMC11898581; doi:10.1128/mbio.02304-24)
Supplement: Supplemental legend — Legend for Fig. S1. [file mbio.02304-24-s0002.docx]

**Supplementary Figure S1. Life cycle of *Plasmodium spp.* highlighting new cryptic erythrocytic stages.** During a mosquito blood meal, female malaria-infected mosquitoes inject sporozoites into the bloodstream, initiating the pre-erythrocytic cycle by entering hepatocytes. Within the liver, *P. vivax* differentiates into either hypnozoites, causing relapses upon reactivation or, similar to *P. falciparum*, into tissue schizonts, releasing merozoites into the bloodstream through merosomes starting the erythrocytic cycle. During this cycle, responsible for the pathology associated with malaria, *P. vivax* merozoites predominantly invade reticulocytes whereas *P. falciparum* has a preference for mature RBCs. Some parasites differentiate into gametocytes, enabling continued transmission. Cryptic erythrocytic infections in the spleen and bone marrow, directly from merozoite invasion or via infected red blood cells, are novel aspects of the life cycle. Circulating gametocytes initiate the sexual cycle in mosquitoes, culminating in sporozoite invasion of salivary glands, completing the complex life cycle across hosts and cell types.
